# Gastrointestinal Microbial Ecology of Weaned Piglets Fed Diets with Different Levels of Glyphosate

**DOI:** 10.1128/spectrum.00615-23

**Published:** 2023-06-15

**Authors:** Sundas Rani, Martin Tang Sørensen, Jordi Estellé, Samantha Joan Noel, Natalja Nørskov, Uffe Krogh, Leslie Foldager, Ole Højberg

**Affiliations:** a Department of Animal and Veterinary Sciences, Aarhus University, Tjele, Denmark; b GABI, INRAE, AgroParisTech, Université Paris-Saclay, Jouy-en-Josas, France; c Bioinformatics Research Centre, Aarhus University, Aarhus, Denmark; University of Texas at San Antonio

**Keywords:** glyphosate, microbial ecology, gastrointestinal tract, piglets

## Abstract

Glyphosate possesses antimicrobial properties, and the present study investigated potential effects of feed glyphosate on piglet gastrointestinal microbial ecology. Weaned piglets were allocated to four diets (glyphosate contents [mg/kg feed]: 0 mg/kg control [CON; i.e., basal diet with no glyphosate added], 20 mg/kg as Glyphomax commercial herbicide [GM_20_], and 20 mg/kg [IPA_20_] and 200 mg/kg [IPA_200_] as glyphosate isopropylamine [IPA] salt). Piglets were sacrificed after 9 and 35 days of treatment, and stomach, small intestine, cecum, and colon digesta were analyzed for glyphosate, aminomethylphosphonic acid (AMPA), organic acids, pH, dry matter content, and microbiota composition. Digesta glyphosate contents reflected dietary levels (on day 35, 0.17, 16.2, 20.5, and 207.5 mg/kg colon digesta, respectively). Overall, we observed no significant glyphosate-associated effects on digesta pH, dry matter content, and—with few exceptions—organic acid levels. On day 9, only minor gut microbiota changes were observed. On day 35, we observed a significant glyphosate-associated decrease in species richness (CON, 462; IPA_200_, 417) and in the relative abundance of certain *Bacteroidetes* genera: CF231 (CON, 3.71%; IPA_20_, 2.33%; IPA_200_, 2.07%) and g_0.24 (CON, 3.69%; IPA_20_, 2.07%; IPA_200_, 1.75%) in cecum. No significant changes were observed at the phylum level. In the colon, we observed a significant glyphosate-associated increase in the relative abundance of *Firmicutes* (CON, 57.7%; IPA_20_, 69.4%; IPA_200_, 66.1%) and a decrease in *Bacteroidetes* (CON, 32.6%; IPA_20_, 23.5%). Significant changes were only observed for few genera, e.g., g_0.24 (CON, 7.12%; IPA_20_, 4.59%; IPA_200_, 4.00%). In conclusion, exposing weaned piglets to glyphosate-amended feed did not affect gastrointestinal microbial ecology to a degree that was considered actual dysbiosis, e.g., no potential pathogen bloom was observed.

**IMPORTANCE** Glyphosate residues can be found in feed made from genetically modified glyphosate-resistant crops treated with glyphosate or from conventional crops, desiccated with glyphosate before harvest. If these residues affect the gut microbiota to an extent that is unfavorable to livestock health and productivity, the widespread use of glyphosate on feed crops may need to be reconsidered. Few *in vivo* studies have been conducted to investigate potential impact of glyphosate on the gut microbial ecology and derived health issues of animals, in particular livestock, when exposed to dietary glyphosate residues. The aim of the present study was therefore to investigate potential effects on the gastrointestinal microbial ecology of newly weaned piglets fed glyphosate-amended diets. Piglets did not develop actual gut dysbiosis when fed diets, containing a commercial herbicide formulation or a glyphosate salt at the maximum residue level, defined by the European Union for common feed crops, or at a 10-fold-higher level.

## INTRODUCTION

Glyphosate-based herbicides (GBHs) are some of the most widely used herbicides worldwide, with glyphosate [*N*-(phosphonomethyl)glycine)] as the active ingredient. In agricultural settings, GBHs are used pre-sowing to control weeds in fields for cereals and soybeans (1). After the introduction of genetically modified glyphosate-tolerant crops in 1996, the volume of GBHs applied increased dramatically and became the most marketed herbicide, accounting for around 25% of the global herbicide market (2, 3). Further, glyphosate is used as a preharvest crop desiccant, applied 1 to 2 weeks before harvest to promote an even crop maturation (2). The tolerance for glyphosate residues in feed and food stuff has been defined by the European Union (EU) and FAO/WHO as maximum residue levels (MRLs). For example, within the EU, glyphosate MRLs in feed crops are 3 mg/kg for maize/corn grains, 10 mg/kg for rye, wheat, and rapeseed, and 20 mg/kg for barley, oat, and soybeans (4). Overall, the extensive use of GBHs on feed crops has thus led to the exposure of livestock, including their gastrointestinal microbiota, to feed residues of glyphosate and, potentially, its primary degradation metabolite aminomethylphosphonic acid (AMPA) (5–7).

Glyphosate acts by inhibiting a key enzyme (5-enolpyruvylshikimate-3-phosphate synthase [EPSPS]) in the shikimate pathway, present in plants and certain microorganisms, and producing chorismate, the precursor of the aromatic amino acids (AA) phenylalanine, tyrosine, and tryptophan. Due to its microorganism-inhibiting capacity, glyphosate has been patented as an antibiotic (8). Two EPSPS classes (I and II) have been defined (9, 10), in general with high and low glyphosate sensitivity, respectively (11). All plants and many Gram-negative bacteria, including members of *Proteobacteria* (e.g., Salmonella and Escherichia) and *Bacteroidetes*, but also certain Gram-positive bacteria (e.g., *Bifidobacterium*), are reported to harbor class I EPSPS (11, 12). Class II EPSPS has been found not only in Gram-positive *Firmicutes* (e.g., *Bacillus*, Streptococcus, and Staphylococcus) but also in *Proteobacteria* (e.g., Pseudomonas and *Agrobacterium*) (13). Recently, EPSPS classes III and IV were identified and reported to be glyphosate resistant (14); also, a glyphosate-tolerant EPSPS variant was found in a Pseudomonas putida strain isolated from glyphosate-polluted soil (10, 15). However, the connections of EPSPS classes to various microbial taxa are still only scarcely investigated, and many EPSPS variants cannot be allocated to defined classes (14). Moreover, glyphosate sensitivity is not solely dependent on EPSPS class (16). Variations in class I EPSPS, such as single AA mutations and enzyme overexpression (due to the competitive nature of the inhibition mechanism), have been reported to confer glyphosate tolerance (11, 12, 17). Thus, there are reports of relatively high glyphosate tolerance in strains of Escherichia coli and Salmonella spp., both harboring class I EPSPS (18, 19).

Due to the absence of the shikimate pathway in the cells of higher animals, glyphosate has been considered safe to use with no direct effects, such as on livestock (20); however, this does not account for a potential indirect influence of glyphosate on animal (or human) health and performance mediated via changes of the gastrointestinal microbiota (21). An investigation of approximately 950 bacterial genomes from the human microbiome project thus revealed genes, encoding glyphosate-sensitive EPSPS, to be present in approximately half of gut bacteria (22).

Few *in vitro* studies have been conducted to investigate the impact of glyphosate on the gut microbiota. In a study with poultry gut bacterial isolates, MICs of glyphosate (as a commercial GBH) was reported to range from 0.07 to 0.3 mg/mL for several commensals (Bacillus badius [0.15 mg/mL], Bacillus cereus [0.3 mg/mL], Bifidobacterium adolescentis [0.07 mg/mL], and Enterococcus faecalis and Enterococcus faecium [both 0.1 mg/mL]), whereas an MIC of 5.0 mg/mL was reported for potential pathogens such as Clostridium perfringens and Salmonella enterica serovars Enteritidis, Gallinarum, and Typhimurium (18). We observed similar differences in the antimicrobial effects of glyphosate, with relatively high MIC values (>10 mg/mL) for strains of E. coli, S. enterica serovar Enteritidis, and S. enterica serovar Typhimurium and lower MIC values for strains of commensals such as Bifidobacterium adolescentis (0.02 mg/mL), Streptococcus alactolyticus (0.07 mg/mL), and Lactobacillus sobrius (0.82 mg/mL) (23). The research group, reporting glyphosate MIC values for poultry bacterial isolates, further studied the impact of glyphosate on the microbial composition of rumen fluid, incubated at a concentration of up to 1 mg of glyphosate/mL, and showed similar results, with pathogenic species such as Clostridium botulinum being favored by the addition of glyphosate (24). These researchers also observed glyphosate to induce accumulation of botulinum neurotoxin (BoNT) and suggested that glyphosate may have inhibited the growth of bacteria capable of degrading BoNT (24). Likewise, 0.1 mg/mL glyphosate was reported to inhibit the growth of enterococci (E. faecalis) that could otherwise have suppressed the growth of Clostridium botulinum antagonistically (25). An *in vitro* study, investigating a collection of E. coli isolates (238 in total) obtained from livestock samples, reported glyphosate MIC values of pathogenic strains to be higher than for commensal strains and suggested that, in general, pathogenic bacteria may adapt more readily to challenging conditions, such as exposure to glyphosate or antibiotics (19). Overall, the authors concluded that *in vivo* glyphosate exposure of mammals may lead to detrimental gut microbial dysbiosis resulting from the wide range of glyphosate sensitivity among bacteria, thereby having indirect adverse effects on animal health. In addition, a functional bioinformatic analysis of the human core microbiome estimated 12 to 26% of the bacterial species to harbor EPSPS I and were thus potentially glyphosate sensitive, emphasizing the potential of glyphosate to affect the gut microbial ecology (14).

Only a few *in vivo* studies have been conducted so far to investigate the potential effects of glyphosate on animals. A long-term (~2 years) study, conducted with rats exposed to glyphosate (as a commercial GBH) in drinking water (0.0001, 400, and 5,000 ppm), reported a sex-dependent effect on the gut microbiota, where in female rats, the relative abundance of *Bacteroidetes* increased and that of *Firmicutes* decreased, although with no clear dose response (26). In another study, the effect of pure glyphosate and a commercial GBH on the gut microbiome was investigated in a 19-week trial with Sprague-Dawley rats, when administered in drinking water (at levels of 1.75 mg/kg of body weight per day) to dams and pups. Microbiome profiling showed significant differences in bacterial composition for pups only, i.e., an increase in *Bacteroidetes* (*Prevotella*) and a reduction in *Firmicutes* (*Lactobacillus*), indicating potential age dependency of glyphosate effects (27). Likewise, a decrease in the relative abundance of *Firmicutes* (*Lactobacillus*) with a concomitant increase in *Bacteroidetes* and *Tenericutes* was reported in rats after 35 days of glyphosate (*N*-phosphonomethyl glycine) exposure (daily gavage of 500 mg/kg of body weight) (28). Dietary exposure of laying hens to a glyphosate-based herbicide (47 mg/kg feed/day) for 7 weeks, followed by 4 weeks on a control diet, revealed the relative abundance of certain cecal bacteria genera (e.g., *Barnesiella* and *Alloprevotella*) to be permanently affected (decrease and increase, respectively), whereas *Ruminococcus* was only affected (decrease) during glyphosate exposure (29). None of these *in vivo* studies, however, analyzed the actual glyphosate levels of gut digesta. Nielsen et al. (30), on the other hand, gavaged rats daily for 2 weeks with glyphosate (0, 2.5, and 25 mg/kg of body weight), resulting in digesta glyphosate levels up to ~50 mg/kg in the colon for the highest dose, and reported no significant effects on gut microbiota composition (30). The outcome of these studies suggests that *in situ* glyphosate concentrations, as well as the duration of exposure, may influence the resulting *in vivo* effects on the gut microbiota. Therefore, it is crucial to define and test actual exposure levels for specific animal species and age groups to evaluate whether, under these conditions, the gastrointestinal microbial ecology will be affected and may influence animal health and/or performance.

The aim of the present study was therefore to investigate whether glyphosate exposure may affect the gastrointestinal microbial ecology of piglets in the first 5 weeks postweaning and potentially lead to negative health impacts due to microbial dysbiosis and, for example, the development of postweaning diarrhea.

## RESULTS

### Glyphosate and AMPA in feed.

The glyphosate level in each of the four treatment diets was analyzed to be 0.02 (control [CON]), 20.8 (GM_20_ [20 mg/kg glyphosate as Glyphomax]), 22.0 (IPA_20_ [20 mg/kg glyphosate as glyphosate isopropylamine salt]), and 208 mg/kg (IPA_200_ [200 mg/kg glyphosate as glyphosate isopropylamine salt]) and thus close to the planned values of 0, 20, 20, and 200 mg/kg feed, respectively (Tables 1 and 2). The feed AMPA levels were 0.01 (CON), 0.11 (GM_20_), 0.09 (IPA_20_), and 0.69 (IPA_200_) mg/kg feed.

**TABLE 1:** Concentrations of glyphosate and AMPA (planned and analyzed) in the treatment diets

Glyphosate and AMPA (mg/kg)	Treatment^*a*^			
	CON	GM_20_	IPA_20_	IPA_200_
Glyphosate (planned)	0	20	20	200
Glyphosate (analyzed)	0.02	20.8	22.0	208
AMPA (analyzed)	0.01	0.11	0.09	0.69

a Control (CON), 20 mg/kg glyphosate as Glyphomax (GM_20_), 20 mg/kg glyphosate as IPA salt (IPA_20_), and 200 mg/kg glyphosate as IPA salt (IPA_200_).

**TABLE 2:** Basal diet ingredients^*a*^

Ingredient	Content (%)
Wheat, not glyphosate desiccated	65.0
Barley, organic	10.0
Soy bean cake, organic	12.0
Fishmeal	5.6
Potato protein concentrate	3.0
Fat (monoglyceride)	1.3
Mineral, vitamin, amino acid mixture	3.1

a For the chemical composition of the basal diet, see Krogh et al. (37).

### Glyphosate and AMPA in digesta.

The different diet levels of glyphosate and AMPA were reflected in different levels of glyphosate, as well as AMPA, in the digesta of all four gut segments both at day 9 (Table 3) and at day 35 (Table 4). For both days, the concentration of glyphosate increased significantly (*P < *0.001) from the proximal to the distal part of the gut. In addition, significantly higher (*P < *0.001) concentrations of glyphosate were observed at day 35 than at day 9. AMPA concentrations followed a similar trend, but no statistical significance was observed.

**TABLE 3:** Glyphosate and AMPA, pH, DM, SCFA, and lactate in gastrointestinal digesta from four segments^*a*^

Parameter and segment	Treatment				SEM^*b*^	*P* ^*d*^
	CON	GM_20_	IPA_20_	IPA_200_		
Glyphosate (mg/kg)						
Stomach	0.01 (0.003)^*c*^	1.18 (0.282)	2.40 (0.233)	15.3 (3.43)	NA	<0.001***†††###
Small intestine (Si3)	0.01 (0.003)	1.63 (0.605)	2.42 (0.444)	13.7 (3.82)	NA	<0.001***†††
Cecum	0.02 (0.005)	2.84 (0.512)	4.57 (0.680)	35.0 (6.85)	NA	<0.001***†††#
Colon (Co2)	0.05 (0.013)	12.6 (2.09)	12.3 (2.17)	166 (12.9)	NA	<0.001***†††
AMPA (mg/kg)						
Stomach	0.01 (0.001)^*c*^	0.01 (0.003)	0.02 (0.004)	0.17 (0.011)	NA	<0.001***†††
Small intestine (Si3)	<0.01 (0.001)	0.02 (0.001)	0.02 (0.001)	0.14 (0.007)	NA	<0.001**†††
Cecum	0.01 (0.002)	0.06 (0.004)	0.08 (0.005)	0.82 (0.060)	NA	<0.001****†††
Colon (Co2)	0.08 (0.001)	0.52 (0.034)	0.42 (0.025)	4.68 (0.296)	NA	<0.001***†††
DM (%)						
Stomach	21.4	23.0	24.7	22.0	1.140	0.13
Small intestine (Si3)	6.84	7.70	7.19	7.46	0.910	0.89
Cecum	8.63	8.43	11.0	8.96	0.920	0.09
Colon (Co2)	15.0	17.6	16.5	16.9	1.460	0.60
pH						
Stomach	3.02	2.81	3.41	3.16	0.249	0.34
Small intestine (Si3)	6.79	6.81	6.67	6.79	0.088	0.61
Cecum	6.00	5.87	5.88	6.12	0.127	0.32
Colon (Co2)	6.69	6.67	6.69	6.32	0.293	0.71
Acetate (mmol/kg)						
Stomach	17.2	10.0	14.5	9.93	1.750	0.01†
Small intestine (Si3)	3.12	2.53	3.05	3.10	0.460	0.73
Cecum	54.4	50.1	59.9	55.4	4.680	0.47
Colon (Co2)	54.2	50.8	55.1	48.7	3.400	0.37
Propionate (mmol/kg)						
Stomach	6.94	3.68	5.11	4.46	0.860	0.05
Small intestine (Si3)	ND	ND	ND	ND		
Cecum	25.8	24.1	27.3	26.4	2.430	0.76
Colon (Co2)	22.8	22.0	22.2	20.9	1.740	0.84
Butyrate (mmol/kg)						
Stomach	7.68	4.03	7.56	4.94	1.620	0.29
Small intestine (Si3)	<0.01	0.02	<0.01	0.02	0.012	0.62
Cecum	10.9	7.13	12.1	9.91	1.850	0.13
Colon (Co2)	9.90	8.75	11.7	9.47	1.180	0.25
Isobutyrate (mmol/kg)						
Stomach	0.03	0.01	<0.01	0.01	0.010	0.10
Small intestine (Si3)	ND	ND	ND	ND		
Cecum	0.38	0.31	0.33	0.43	0.080	0.66
Colon (Co2)	1.01	1.14	0.87	1.22	0.160	0.37
Valerate (mmol/kg)						
Stomach	2.54	1.41	2.39	1.94	0.630	0.55
Small intestine (Si3)	ND	ND	ND	ND		
Cecum	2.29	1.46	2.13	2.06	0.550	0.58
Colon (Co2)	2.35	2.18	2.23	2.66	0.480	0.84
Isovalerate (mmol/kg)						
Stomach	0.04	0.01	<0.01	0.02	0.010	0.04*
Small intestine (Si3)	ND	ND	ND	ND		
Cecum	0.13	0.10	0.16	0.24	0.050	0.27
Colon (Co2)	0.58	0.55	0.48	0.78	0.140	0.44
Lactate (mmol/kg)						
Stomach	39.8	28.9	33.5	20.7	5.900	0.11
Small intestine (Si3)	23.8	20.0	26.0	18.5	5.880	0.73
Cecum	4.29	13.1	9.80	0.84	5.600	0.41
Colon (Co2)	0.77	5.93	0.28	0.21	3.000	0.38

a Control (CON), 20 mg/kg glyphosate as Glyphomax (GM_20_), 20 mg/kg glyphosate as IPA (IPA_20_), and 200 mg/kg glyphosate as IPA (IPA_200_). Samples were obtained from piglets sacrificed on day 9 of treatment. The data are presented as EM-means (estimated marginal means; *n* = 13 piglets per treatment). NA, not applicable; ND, not detectable.

b Average of standard errors of means.

c Due to the heterogeneity of glyphosate and AMPA data, the standard error of individual means is presented in parenthesis instead of average SEM (see also Materials and Methods).

d *, *P*_adj_ < 0.05; **, *P*_adj_ < 0.01; ***, *P*_adj_ < 0.001 (effect of 20 mg/kg glyphosate, contrast CON versus IPA_20_). †, *P*_adj_ < 0.05; ††, *P*_adj_ < 0.01; †††, *P*_adj_ < 0.001 (effect of 200 mg/kg glyphosate, contrast CON versus IPA_200_). #, *P*_adj_ < 0.05; ##, *P*_adj_ < 0.01; ###, *P*_adj_ < 0.001 (effect of Glyphomax additives, contrast GM_20_ versus IPA_20_).

**TABLE 4:** Glyphosate and AMPA, pH, DM, SCFA, and lactate in gastrointestinal digesta from four segments^*a*^

Parameter and segment	Treatment				SEM^*b*^	*P* ^*d*^
	CON	GM20	IPA20	IPA200		
Glyphosate (mg/kg)						
Stomach	0.02 (0.008)^*c*^	2.12 (0.351)	1.63 (0.452)	21.0 (3.19)	NA	<0.001***†††
Small intestine (Si3)	0.04 (0.009)	6.91 (0.745)	6.04 (0.897)	81.4 (10.71)	NA	<0.001***†††
Cecum	0.04 (0.009)	4.97 (0.605)	5.24 (0.437)	64.9 (5.95)	NA	<0.001***†††
Colon (Co2)	0.17 (0.043)	16.2 (0.98)	20.5 (1.84)	207 (16.8)	NA	<0.001***†††
AMPA (mg/kg)						
Stomach	0.01 (0.001)^*c*^	0.01 (0.003)	0.01 (0.002)	0.11 (0.013)	NA	<0.001†††
Small intestine (Si3)	0.01 (0.001)	0.06 (0.007)	0.05 (0.008)	0.42 (0.048)	NA	<0.001***†††
Cecum	0.01 (0.001)	0.11 (0.015)	0.12 (0.013)	1.00 (0.131)	NA	<0.001***†††
Colon (Co2)	0.02 (0.002)	0.54 (0.054)	0.71 (0.113)	5.69 (0.849)	NA	<0.001†††
DM (%)						
Stomach	22.90	24.10	22.90	23.30	1.580	0.86
Small intestine (Si3)	11.40	12.60	12.20	13.10	0.780	0.30
Cecum	11.90	12.10	12.00	12.00	0.490	0.98
Colon (Co2)	19.70	19.50	19.60	19.60	1.040	0.94
pH						
Stomach	3.48	3.56	3.59	3.30	0.285	0.80
Small intestine (Si3)	6.55	6.44	6.57	6.29	0.108	0.12
Cecum	5.95	5.76	5.87	5.77	0.081	0.19
Colon (Co2)	6.39	6.50	6.39	6.48	0.097	0.74
Acetate (mmol/kg)						
Stomach	7.88	8.04	5.57	5.85	1.460	0.44
Small intestine (Si3)	5.85	7.82	5.66	5.85	0.844	0.05#
Cecum	70.50	69.50	68.10	68.50	2.880	0.91
Colon (Co2)	62.10	64.20	61.60	65.50	2.350	0.18
Propionate (mmol/kg)						
Stomach	3.75	4.03	1.51	2.29	0.990	0.19
Small intestine (Si3)	0.03	0.13	0.05	0.01	0.030	0.08
Cecum	25.80	24.10	27.30	26.40	2.460	0.67
Colon (Co2)	25.50	26.70	22.90	28.10	1.750	0.12
Butyrate (mmol/kg)						
Stomach	2.18	1.24	0.69	0.79	0.604	0.20
Small intestine (Si3)	0.11	0.33	0.21	0.17	0.081	0.18
Cecum	14.30	15.80	14.60	15.30	1.140	0.71
Colon (Co2)	14.80	16.50	17.60	19.40	1.290	0.04††
Isobutyrate (mmol/kg)						
Stomach	1.55	1.38	1.38	1.61	0.130	0.44
Small intestine (Si3)	ND	ND	ND	ND		
Cecum	0.49	0.34	0.41	0.47	0.070	0.44
Colon (Co2)	1.55	1.38	1.38	1.61	0.130	0.44
Valerate (mmol/kg)						
Stomach	0.76	0.33	0.16	0.12	0.260	0.27
Small intestine (Si3)	ND	ND	ND	ND		
Cecum	1.62	2.14	2.00	2.65	0.330	0.13
Colon (Co2)	2.48	2.63	2.90	4.22	0.290	<0.001†††
Isovalerate (mmol/kg)						
Stomach	ND	ND	ND	ND		
Small intestine (Si3)	ND	ND	ND	ND		
Cecum	0.15	0.23	0.21	0.21	0.070	0.88
Colon (Co2)	1.00	0.70	0.80	1.09	0.100	0.18
Lactate (mmol/kg)						
Stomach	15.70	15.30	12.80	12.80	4.910	0.88
Small intestine (Si3)	25.70	25.30	25.50	33.40	6.470	0.68
Cecum	0.77	1.23	0.66	5.60	2.700	0.47
Colon (Co2)	0.10	0.06	0.10	0.01	0.088	0.81

a Data are presented as EM-means (estimated marginal means; *n* = 13 piglets per treatment). Control (CON), 20 mg/kg glyphosate from Glyphomax (GM_20_), 20 mg/kg glyphosate as IPA (IPA_20_), and 200 mg/kg glyphosate as IPA (IPA_200_). Samples were obtained from piglets sacrificed on day 35 of treatment. NA, not applicable; ND, not detectable.

b Average of standard errors of means.

c Due to the heterogeneity of glyphosate and AMPA data, the standard error of individual means is presented in parenthesis instead of average SEM (see also Materials and Methods).

d *, *P*_adj_ < 0.05; **, *P*_adj_ < 0.01; ***, *P*_adj_ < 0.001 (effect of 20 mg/kg glyphosate, contrast CON versus IPA_20_). †, *P*_adj_ < 0.05; ††, *P*_adj_ < 0.01; †††, *P*_adj_ < 0.001 (effect of 200 mg/kg glyphosate, contrast CON versus IPA_200_). #, *P*_adj_ < 0.05; ##, *P*_adj_ < 0.01; ###, *P*_adj_ < 0.001 (effect of Glyphomax additives, contrast GM_20_ versus IPA_20_).

### Digesta DM, pH, SCFA, and lactate.

The digesta short-chain fatty acid (SCFA) and lactate concentrations, dry matter (DM) content, and pH are shown in Table 3 (day 9) and Table 4 (day 35). The results are presented only for treatment effects. We analyzed the effect of sex, as well as the treatment and sex interactions, but no significant effects were found (*P > *0.05).

**(i) Day 9.** The DM mean levels across treatments were 23.0% (stomach), 7.3% (small intestine), 9.2% (cecum), and 16.5% (Colon) and not affected by treatment in any of the gut segments (Table 3).

Likewise, we found no significant treatment effects on digesta pH with mean values of 3.10 (stomach), 6.77 (small intestine), 5.97 (cecum), and 6.59 (colon) (Table 3).

Among the SCFAs, acetate in stomach digesta was highest for control, and the reduction for the IPA_200_ treatment was significant (*P*_adj_ < 0.05); similarly, the isovalerate level was highest in stomach digesta, and the reduction for the IPA_20_ treatment was significant (*P*_adj_ < 0.05). We observed no significant effects of treatments on digesta SCFA levels in the small intestine, cecum, and colon; also, the lactate levels were not affected by treatment in any of the gut segments (Table 3).

**(ii) Day 35.** Compared to day 9, we observed numerically higher digesta DM levels at day 35, with mean values across treatments of 23.3% (stomach), 12.3% (small intestine), 12.0% (cecum), and 19.6% (colon), but, as for day 9, we observed no significant effects of treatment (*P > *0.05) at day 35 (Tables 3 and 4).

The mean values of digesta pH at day 35 across treatments were 3.46 (stomach), 6.47 (small intestine), 5.75 (cecum), and 6.25 (colon) (Table 4). Thus, stomach pH was higher in samples at day 35 compared to day 9, whereas for the three other segments it was lower (Tables 3 and 4). No significant effect of treatment was observed for the digesta pH (*P > *0.05).

Stomach SCFA and lactate levels were lower at day 35 compared to day 9 (Tables 3 and 4). For the three other gut segments, SCFA levels were higher compared to day 9. The lactate level was likewise higher in the small intestine at day 9; however, there was no clear pattern in the cecum and colon. In the stomach and cecum, we observed no effects of treatment on SCFA and lactate. However, compared to the control, significantly higher levels of digesta butyrate (*P*_adj_ < 0.01) and valerate (*P*_adj_ < 0.001) were observed in the colon for the IPA_200_ treatment. In addition, the acetate level in small intestine digesta was higher for GM_20_ than for IPA_20_ (*P*_adj_ < 0.05).

### Microbiota diversity.

Across all samples, a total of 1,016 operational taxonomic units (OTUs; species level) was obtained after filtering out the low-abundance OTUs (0.005% threshold). Overall, the complete data set (across segments, treatments, and sampling days) revealed no significant effects of treatment on the alpha-diversity (richness and Shannon index) and beta-diversity (Whittaker index) (data not shown). Microbial diversity was not affected by treatment on day 9 in any of the four gut segments (Table 5). However, on day 35, we observed a significant decrease in species richness in the cecum (*P*_adj_ < 0.05) for the IPA_200_ treatment compared to CON. In addition, a significantly (*P*_adj_ < 0.05) higher beta-diversity (Whittaker index) was observed in the small intestine for the IPA_20_ compared to the GM_20_ treatment (Table 5). Overall, the data set demonstrated significant (*P < *0.001) effects of segment on alpha-diversity (richness and Shannon index) and beta-diversity (Bray-Curtis dissimilarities) across all treatments and both sampling days (see Fig. S1 in the supplemental material). A comparison of sampling days (day 9 and 35) for cecum parameters showed a significant (*P < *0.001) increase in richness and Shannon index and a decrease in the beta-diversity (Whittaker index) (see Table S1). The effect of sampling day on colon parameters showed a significant (*P < *0.001) increase in richness and the Shannon index, as well as in the Whittaker index (see Table S1). For the stomach, we observed significant (*P = *0.01) decrease in richness and an increase in Whittaker index from days 9 to 35, and for the small intestine we observed increase in richness (*P = *0.05) and the Shannon index (*P < *0.01); no effect (*P > *0.05) was observed for the Shannon index in the stomach and the Whitaker index in the small intestine (see Table S1). We observed no significant effects of sex on the diversity parameters (see Fig. S2).

**TABLE 5:** Microbial diversity in gastrointestinal digesta from piglets, sacrificed on day 9 or day 35 of treatment^*a*^

Day and diversity index	Diversity type	Segment	Treatment^*b*^				SEM^*c*^	*P* ^*d*^
			CON	GM_20_	IPA_20_	IPA_200_		
Day 9								
Alpha	Richness	Stomach	227	217	234	227	10.4	0.73
		Small intestine (Si3)	132	129	134	133	10.9	0.98
		Cecum	422	408	412	375	18.5	0.30
		Colon (Co2)	451	424	428	410	15.2	0.29
	Shannon	Stomach	3.25	3.21	3.30	3.36	0.09	0.70
		Small intestine (Si3)	1.91	1.59	1.70	1.79	0.17	0.59
		Cecum	4.38	4.28	4.34	4.15	0.12	0.56
		Colon (Co2)	4.66	4.57	4.61	4.49	0.08	0.54
Beta	Whittaker	Stomach	0.42	0.42	0.40	0.41	0.01	0.63
		Small intestine (Si3)	0.46	0.51	0.47	0.48	0.01	0.26
		Cecum	0.39	0.41	0.40	0.43	0.01	0.14
		Colon (Co2)	0.36	0.37	0.37	0.39	0.01	0.16
Day 35								
Alpha	Richness	Stomach	290	251	267	262	24.0	0.70
		Small intestine (Si3)	146	145	161	144	13.2	0.78
		Cecum	462	453	442	417	12.6	0.08†
		Colon (Co2)	486	489	472	449	12.0	0.09
	Shannon	Stomach	3.40	3.28	3.05	3.20	0.11	0.17
		Small intestine (Si3)	2.03	2.17	2.16	2.11	0.13	0.86
		Cecum	4.68	4.56	4.54	4.41	0.08	0.17
		Colon (Co2)	4.83	4.80	4.78	4.66	0.06	0.26
Beta	Whittaker	Stomach	0.43	0.42	0.45	0.44	0.01	0.18
		Small intestine (Si3)	0.47	0.45	0.50	0.48	0.01	0.06#
		Cecum	0.32	0.32	0.32	0.34	0.01	0.22
		Colon (Co2)	0.30	0.29	0.30	0.31	0.01	0.49

a The data are presented as EM-means (estimated marginal means; *n* = 13 piglets per treatment).

b Control (CON), 20 mg/kg glyphosate as Glyphomax (GM_20_), 20 mg/kg glyphosate as IPA salt (IPA_20_), 200 mg/kg glyphosate as IPA salt (IPA_200_).

c Average of standard errors of means.

d †, *P*_adj_ < 0.05; ††, *P*_adj_ < 0.01; †††, *P*_adj_ < 0.001 (effect of 200 mg/kg of glyphosate, contrast CON versus IPA_200_). #, *P*_adj_ < 0.05; ##, *P*_adj_ < 0.01; ###, *P*_adj_ < 0.001 (effect of Glyphomax additives, contrast GM_20_ versus IPA_20_).

### Microbiota composition.

**FIG 1:**
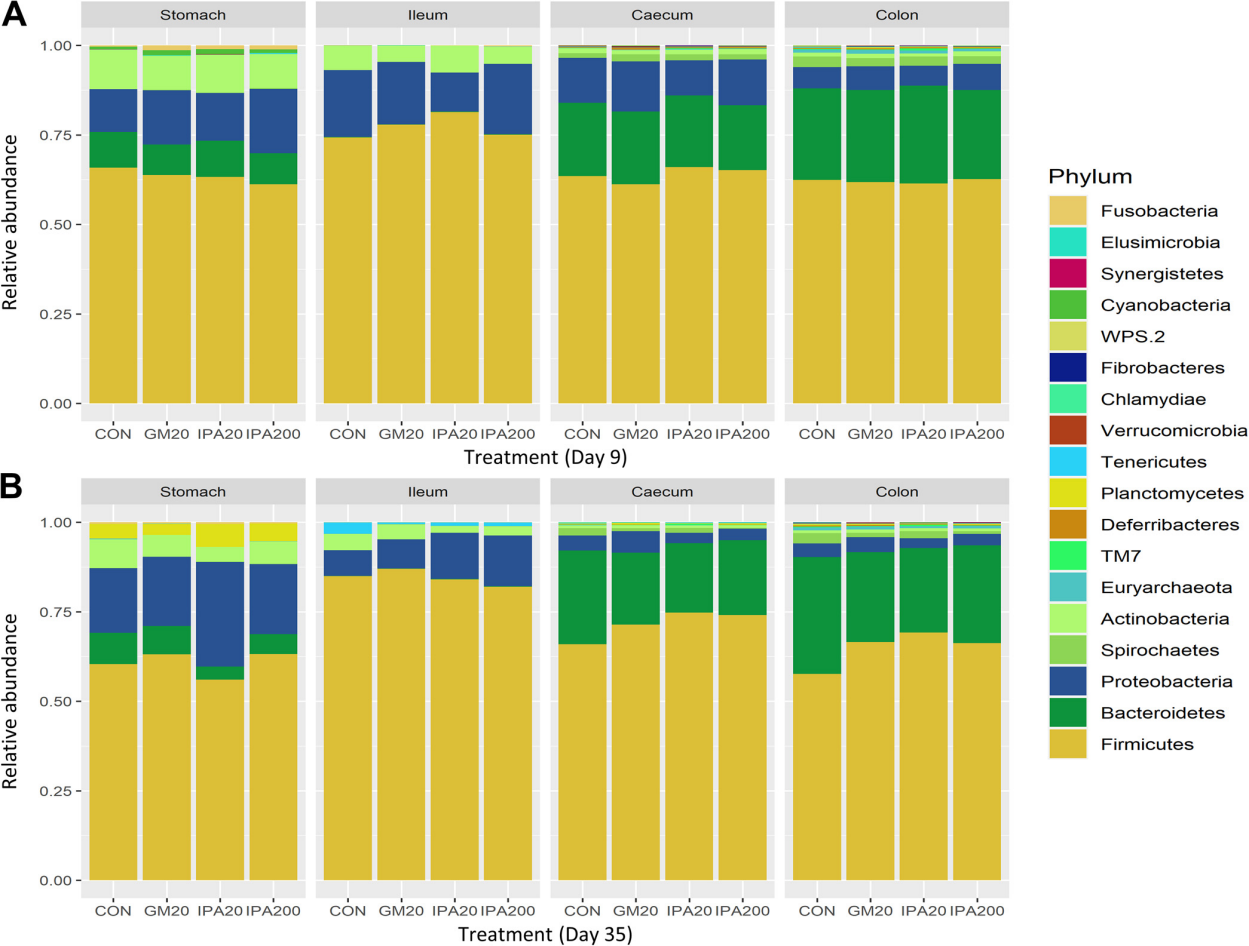
Microbiota composition (phylum level) in digesta from stomach, small intestine (Si3), cecum and colon (Co2), illustrated by the relative abundance of the 18 observed phyla on day 9 (A) and day 35 (B) in treatment. Treatments: Control (CON), 20 mg/kg glyphosate as Glyphomax (GM_20_), and 20 mg/kg glyphosate as IPA salt (IPA_20_), 200 mg/kg glyphosate as IPA salt (IPA_200_).

**FIG 2:**
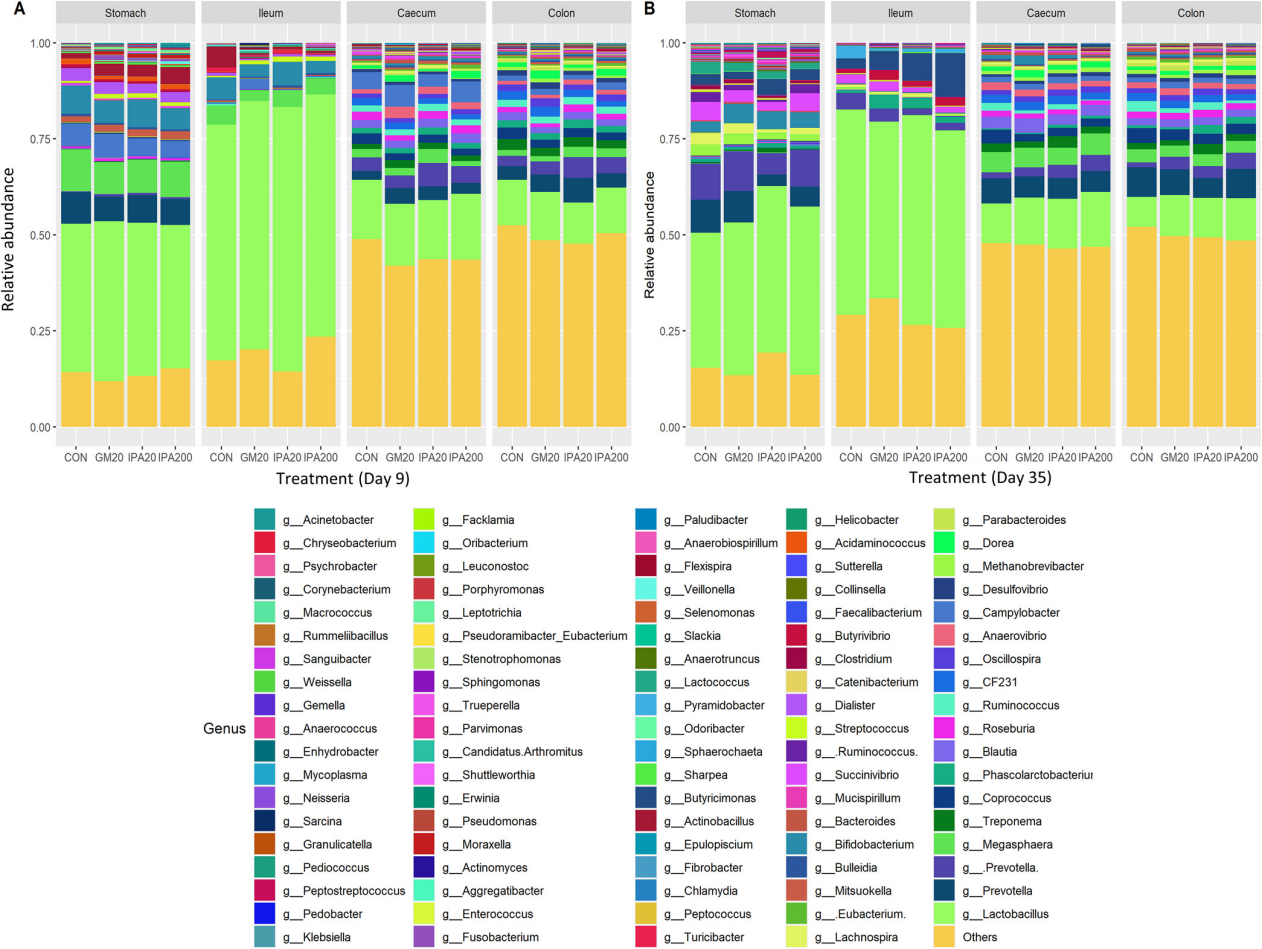
Microbiota composition (genus level) in digesta from stomach (Sto), small intestine (Si3), cecum and colon (Co2), illustrated by the relative abundance of selected genera on day 9 (A) and day 35 (B) in treatment. Treatments: Control (CON), 20 mg/kg glyphosate as Glyphomax (GM_20_), 20 mg/kg glyphosate as IPA salt (IPA_20_), and 200 mg/kg glyphosate as IPA salt (IPA_200_).

Overall, the analysis of all samples revealed 18 phyla and 101 genera (Fig. 1 and 2, respectively) in the gut digesta across sampling days, with *Firmicutes*, *Proteobacteria*, *Bacteroidetes*, *Actinobacteria*, and *Spirochaetes* as the dominant phyla. The relative abundances of selected phyla and genera are presented in Table 6 (see also Tables S2 and S3 in the supplemental material).

**TABLE 6:** Relative abundance of a selected set of bacterial phyla and genera in gastrointestinal digesta from piglets, sacrificed on day 9 or day 35 of treatment^*a*^

Day and segment	Taxon (phylum, genus)	Treatment^*b*^				SEM^*c*^	*P* ^*d*^
		CON	GM_20_	IPA_20_	IPA_200_		
Day 9							
Stomach							
	*Firmicutes*	65.7	63.9	63.3	61.2	4.58	0.92
	f_*Clostridiaceae*; g_	0.02	0.25	0.04	0.11	0.05	0.03#
	*Proteobacteria*	11.9	15.2	13.3	18.0	3.63	0.66
	f_*Enterobacteriaceae*; g_0.5	0.07	0.30	0.33	1.15	0.31	0.08
							
							
Small intestine (Si3)							
	*Cyanobacteria*	0.04	0.01	0.01	0.01	0.01	0.09
							
Cecum							
	*Firmicutes*	63.8	61.1	65.9	65.0	4.00	0.83
	f_*Ruminococcaceae*; g_0.38	15.6	11.0	13.2	14.2	1.12	0.04
	*Bacteroidetes*	20.4	20.3	20.0	18.2	2.48	0.89
	f_ *Paraprevotellaceae*; g_[*Prevotella*] 2	3.64	3.27	6.15	4.36	0.80	0.06#
	*Cyanobacteria*	0.03	0.19	0.01	0.03	0.04	0.03#
							
Day 35							
Stomach							
	*Bacteroidetes*	8.82	7.88	3.79	5.50	1.62	0.12
	g_*Prevotella* 1	8.68	8.09	3.26	5.15	1.72	0.10
							
Small intestine (Si3)							
	*Firmicutes*	84.7	87.2	83.8	82.2	3.41	0.76
	g_*Ruminococcus*	0.01	0.10	0.07	0.02	0.01	0.02*#
	g_*Roseburia*	0.01	0.01	0.04	0.01	0.01	0.04#
							
Cecum							
	*Firmicutes*	66.0	71.3	75.0	74.0	2.84	0.13
	f_*Ruminococcaceae*; g_0.38	13.0	15.3	15.0	16.4	1.01	0.10
	g_*Megasphaera*	1.62	2.34	3.44	4.28	0.83	0.13
	*Bacteroidetes*	26.1	20.1	19.2	20.9	2.15	0.11
	f_*Paraprevotellaceae*; g_CF231	3.71	2.02	2.33	2.07	0.40	0.01*†
	o_*Bacteroidales* 2; g_0.24	3.69	1.89	2.07	1.75	0.42	0.01*†
	*Spirochaetes*						
	g_*Treponema*	2.05	0.91	1.27	0.29	0.48	0.08†
							
Colon (Co2)							
	*Firmicutes*	57.7	66.5	69.4	66.1	2.53	0.01**†
	g_*Megasphaera*	1.37	3.30	3.16	4.39	0.78	0.06
	g_Streptococcus	0.76	0.57	2.32	1.79	0.51	0.05#
	*Bacteroidetes*	32.6	25.1	23.5	27.4	2.03	0.01*
	f_*Paraprevotellaceae;* g_CF231	4.07	2.89	2.79	2.76	0.43	0.11
	o_*Bacteroidales* 2; g_0.24	7.12	4.56	4.59	4.00	0.66	0.01*†
	o_*Bacteroidales*; f_RF16; g_0.17	0.97	0.28	0.42	0.22	0.20	0.05†

a Data are presented as EM-means (estimated marginal means; *n* = 13 piglets per treatment). For a more comprehensive data set, see Table S2 and S3 in the supplemental material.

b Control (CON), 20 mg/kg glyphosate as Glyphomax (GM_20_), 20 mg/kg glyphosate as IPA salt (IPA_20_), and 200 mg/kg glyphosate as IPA salt (IPA_200_).

c Average of standard errors of means.

d *, *P*_adj_ < 0.05; **, *P*_adj_ < 0.01; ***, *P*_adj_ < 0.001 (effect of 20 mg/kg of glyphosate, contrast CON versus IPA_20_). †, *P*_adj_ < 0.05; ††, *P*_adj_ < 0.01; †††, *P*_adj_ < 0.001 (effect of 200 mg/kg of glyphosate, contrast CON versus IPA_200_). #, *P*_adj_ < 0.05; ##, *P*_adj_ < 0.01; ###, *P*_adj_ < 0.001 (effect of Glyphomax additives, contrast GM_20_ versus IPA_20_).

**(i) Day 9.** In all four segments, *Firmicutes* were found to be the most dominant phylum, whereas the order of abundance for the other major phyla varied across segments (Fig. 1A; see also Table S2). Stomach and small intestine followed the same order of abundance, with mean values in small intestine of *Firmicutes* (77.1%), followed by *Proteobacteria* (17.5%), *Actinobacteria* (8.0%), and *Bacteroidetes* (5.0%). In the colon, the mean values of *Firmicutes* (62.1%) were followed by *Bacteroidetes* (23.5%), *Proteobacteria* (10.5%), *Spirochaetes* (2.5%), and *Actinobacteria* (1.5%), with the same order of abundance in the cecum.

At the phylum level, the relative abundances were in general not observed to be affected by treatment, except for the low-abundance (<0.2%) *Cyanobacteria* phylum that in the cecum was significantly (*P*_adj_ < 0.05) higher for GM_20_ compared to IPA_20_ (Table 6; see also Table S2).

Overall, the majority of the genera were not significantly affected by treatment (Fig. 2A; see also Table S2). The low-abundance (below 0.5%) *Clostridiaceae* genus (g_) was significantly higher (*P*_adj_ < 0.05) in the stomach for GM_20_ compared to IPA_20_ (Table 6). In the cecum, a relatively high-abundance (above 3%) *Paraprevotellaceae* genus (g_[*Prevotella*]) was significantly lower (*P*_adj_ < 0.05) for GM_20_ compared to IPA_20_ (Table 6).

**(ii) Day 35.** As observed on day 9, *Firmicutes* was the most abundant phylum on day 35 in all segments, but the order of abundance differed along the gut for the other major phyla (Fig. 1B; see Table S3). In the stomach, *Firmicutes* (61%) was followed by *Proteobacteria* (22%), *Bacteroidetes* (7%), and *Actinobacteria* (6.1%). In the small intestine, the order of abundance was *Firmicutes* (84.4%), *Proteobacteria* (11%), *Actinobacteria* (3.1%), and *Bacteroidetes* (0.1%), while in the colon *Firmicutes* (65%) was followed by *Bacteroidetes* (27.2%), *Proteobacteria* (4%), and *Actinobacteria* (0.2%), with the same order of abundance in the cecum. For some phyla, the relative abundances were affected by treatment on day 35, e.g., with a decrease in *Bacteroidetes* in the stomach and cecum for glyphosate treatments compared to CON, and a significant reduction in the colon (*P*_adj_ < 0.01) was observed for IPA_20_ compared to CON. For *Firmicutes*, we observed a significantly greater relative abundance in the colon for IPA_20_ (*P*_adj_ < 0.01) and IPA_200_ (*P*_adj_ < 0.05) compared to CON. The levels of *Spirochaetes* were significantly lower (*P*_adj_ < 0.05) in the cecum for IPA_200_ compared to CON (Table 6) and showed a numerical decrease in the colon for all treatments compared to CON (see Table S3).

In the cecum, the observed effect on *Bacteroidetes* was mainly a significant lower relatively abundance for IPA_20_ (*P*_adj_ < 0.05) and IPA_200_ (*P*_adj_ < 0.05) compared to the CON of two genera, namely, CF231 and unidentified *Bacteroidales* genus g_24, both with relatively low abundances (1.75 to 3.71%) (Table 6). In the colon, a significant decrease (*P*_adj_ < 0.05) was observed for the less-dominant Streptococcus (an approximately 0.5 to 2.5% abundance) for GM_20_ compared to IPA_20_. Further, compared to CON, a significant treatment-associated decrease in the dominant (>4%) *Bacteroidetes* genus g_24 was observed for IPA_20_ (*P*_adj_ < 0.05) and IPA_200_ (*P*_adj_ < 0.05). Similarly, the less-dominant (<1%) genus g_17 was decreased significantly (*P*_adj_ < 0.05) for IPA_200_ compared to CON (Table 6).

In summary, all gut segments were dominated by *Firmicutes*, and their relative abundance increased from day 9 to day 35 in the cecum and colon, whereas *Proteobacteria* and *Bacteroidetes* decreased.

### Analysis of predicted metabolic functions.

The PICRUSt package and the KEGG database were used to infer associations between the bacterial taxa and metabolic functions (see Fig. S3). OTU-based analysis revealed none of the predicted metabolic functions to be significantly affected by glyphosate treatment. However, the predicted functions were significantly affected by day (*P < *0.001), gut segment (*P < *0.001), and sex (*P = *0.05).

## DISCUSSION

To obtain a glyphosate-free control diet, we used selected raw materials expected not to have been exposed to glyphosate, such as nondesiccated wheat and organically grown barley and soybeans. The two included glyphosate levels (20 and 200 mg/kg) were obtained by amending the control diet either with pure glyphosate in the form of the IPA salt, commonly used in commercial GBHs due to its relatively high solubility (31) or as a commercial GBH (Glyphomax). The analysis of the final feed mixtures revealed the glyphosate content of our control diet to be close to zero, but not completely glyphosate free. The glyphosate levels of the other treatment diets were close to the planned values outlined above. We are aware of that in practice, a glyphosate concentration of 20 mg/kg (the maximum residue level [MRL] for many commonly used feed crops) may rarely be found in full feed mixtures. Glyphosate is commonly detected but is usually present well below MRL, as shown by a study investigating the glyphosate in cereals produced in Denmark during the years 1997 to 1999 (32), as well as a later Danish survey, for the years 2008 to 2013, revealed wheat and barley samples to have glyphosate residues up to 4.1 and 13 mg/kg, respectively (33). Still, we chose to include a glyphosate level of 20 mg/kg, representing a maximum legal level for many common crops, as well as a 10-fold higher level.

We analyzed the digesta levels of glyphosate and AMPA, to which the gut microbiota was exposed *in vivo*. When animals ingest glyphosate-containing feed, the glyphosate may be absorbed, diluted (water intake, intestinal secretions), concentrated (water resorption in the colon), and potentially degraded, e.g., to AMPA, all affecting the actual glyphosate concentrations to be reached in the lumen digesta. The data of the present study demonstrated that digesta glyphosate levels increased from the proximal to the distal part of the gut, reaching levels in the colon similar to that of the respective diets. We further observed digesta glyphosate concentrations to increase from day 9 to day 35, and more than could be accounted for by the concomitant increase in digesta DM (Tables 3 and 4), but we have no clear explanation for this observation. It is also important to emphasize that the *in vivo* digesta glyphosate concentrations, obtained via the treatment diets, reached levels up to ~0.2 mg/mL. These levels are similar to the MIC values (0.075 to 0.150 mg/mL) reported for some of the most glyphosate-sensitive poultry gut bacteria, like species of *Enterococcus*, *Bacillus*, and *Bifidobacterium* by Shehata et al. (18). Moreover, it is evident and we have observed that the growth rate of bacterial strains may be significantly affected by lower concentrations (defined as minimum effective concentration) than the observed MIC values (23), indicating that glyphosate-associated changes of the gut microbiota could potentially be expected under the *in vivo* conditions of the present study.

The digesta glyphosate levels in the colon, as we report here, are close to the glyphosate concentrations we previously estimated, taking some of the above-mentioned gastrointestinal factors (absorption, dilution, and water resorption) into consideration (23). For these estimates, we did not take glyphosate degradation to AMPA into account, since it is pertinent to stress that no glyphosate-degrading gut microbes have been identified. The data of the present study shows digesta AMPA concentrations reaching 3 to 4% of the digesta glyphosate levels (colon, day 35), whereas the feed AMPA concentrations were only ~0.5% of the glyphosate levels. However, we cannot conclude whether this proportional shift is due to differentiated absorption of the two compounds, potential degradation of glyphosate, e.g., to AMPA, or a combination of both. A study analyzing 2,000 publicly available human gut microbiomes (metagenomes) for the presence of genes involved in glyphosate metabolism did not detect the degradation pathway to AMPA in the gut microbiomes, although glyphosate degradation to AMPA was reported for soil microbes (34, 35). The literature regarding potential effects of AMPA on gut microorganism is scarce; however, a study investigating the *in vivo* effects of glyphosate and AMPA on bee gut microbiota reported glyphosate-associated changes but no apparent *in vivo* effects of AMPA. The authors thus argued that degradation to AMPA, either before or after ingestion, could alleviate otherwise detrimental glyphosate effects (36).

As we reported previously (37), feed intake and weight gain of the animals in the present study were overall not observed to be significantly affected by glyphosate treatment. In addition to the fecal score, reported as a potential indicator of diarrhea-like conditions in Krogh et al. (37), we report here that the digesta DM content is (numerically) lower on day 9 than on day 35, but we observed no effect of glyphosate treatment. These observations indicate that the glyphosate exposure (levels and exposure time) of the present study neither affected animal performance nor induced, e.g., the bloom of pathogens such as enterotoxigenic E. coli, as might otherwise have been expected from the observations reported by Bote et al. (19).

In the present study, significantly lower concentrations of acetate and propionate were observed in the stomach on day 9 for animals exposed to glyphosate; the acetate/propionate ratio was not affected. Nielsen et al. (30) observed significantly reduced acetate concentrations in cecal samples from glyphosate-treated rats, as well as a glyphosate-associated pH increase in digesta and feces. In addition, another study reported that the pathways for acetate production were similar among a wide group of bacteria, whereas butyrate, propionate, and lactate production pathways varied more among bacteria and also depended on the type of substrate being metabolized (38). Acetate and propionate have been reported mainly to be produced by *Bacteroidetes*; however, the major producers of butyrate have commonly been reported as *Firmicutes* (39, 40). Nevertheless, we did not observe the relative abundance of *Bacteroidetes* to be significantly affected on day 9. Moreover, a treatment-associated increase in butyrate and valerate concentrations in colon digesta on day 35 may reflect the observed increase in the relative abundance of *Firmicutes*, where, for example, the *Megasphaera* genus, which also tended to increase, is known to harbor butyrate-producing species (41). However, Li et al. (41) also reported that butyrate producers could be found among members of the *Bacteroidetes*, a phylum that in our study showed a treatment-associated decrease in relative abundance (day 35, colon). Thus, correlations like this should be interpreted with caution, and it should be emphasized that the actual microorganism levels are not reflected by the relative abundances.

To enhance plant (cell) penetration, glyphosate adjuvants are typically added to GBHs. Some studies have shown toxic and endocrine-disrupting behavior of the adjuvants compared to glyphosate alone (42), and the use of tallow amines as adjuvants in GBHs has been banned recently in the EU because of their reported side effects (3, 43). Overall, we observed only minor differences in the effects of GBH and glyphosate IPA salt such as, for example, on day 35, the significantly lower relative abundance of Streptococcus in colon digesta and the higher concentration of acetate in small intestine digesta for the piglets fed GBH compared to glyphosate IPA. A study, investigating the *in vitro* growth of E. coli in a minimal medium amended with different glyphosate formulations also reported a somewhat similar impact of one GBH product and glyphosate IPA salt; however, another GBH product had a significantly higher impact on E. coli growth, clearly illustrating that commercial GBHs may differ considerably in their antimicrobial properties (30). Likewise, the GBH Roundup GT Plus was observed to inhibit growth of Lactobacillus rhamnosus strains at glyphosate concentrations that did not affect growth of the strains using pure glyphosate or the GBH Roundup MON 52276 (44).

The resilience of gut microbial communities is considered crucial for health and performance of animals (45). The most abundant phyla reported, after high-throughput sequencing technologies were applied, in pig gut samples were *Firmicutes*, *Bacteroidetes*, and *Proteobacteria* (46, 47), similar to the observations of the present study. We observed a glyphosate-associated increase in the relative abundance of *Firmicutes* and a decrease in *Bacteroidetes* in colon and, to a lesser degree, cecum digesta on day 35, which could be explained by members of the two phyla, predominantly harboring the glyphosate-resistant class II EPSPS and the glyphosate-sensitive class I EPSPS, respectively (9–11, 17). However, several *in vivo* rat studies have reported the exact opposite observation, with a glyphosate-associated decrease in relative abundance of *Firmicutes* and a concomitant increase in *Bacteroidetes* (26–28). This discrepancy could illustrate a lack of knowledge concerning the actual EPSPS class being harbored by subtaxa within these phyla (14), as well as the discrepancy in reported observations emphasizes not to draw hasty and simplified conclusions on the potential *in vivo* glyphosate-associated effects on the gut microbiota.

We did not observe any glyphosate-associated change in predicted metabolic functions of the gut microbiota as evaluated by PICRUSt-based analysis. Likewise, a recent study (34) performed a PICRUSt-based reanalysis of the 16S rRNA gene amplicon data of Nielsen et al. (30), and observed no glyphosate-associated changes in the abundance of the shikimate pathway genes, in accordance with the observations of Nielsen et al. that showed no glyphosate-associated effect on the microbiota composition. On the other hand, Mesnage and Antoniou (34) observed glyphosate-associated effect on other metabolic pathways all involving chorismate; since chorismate is the final product of the shikimate pathway, these observations indicate that the shikimate pathway (i.e., EPSPS) activity may have been impaired by glyphosate, reducing the provision of chorismate as a substrate for these other pathways. However, our functional analysis did not reveal a pattern like this.

It has been suggested that, in general, digesta amino acid levels are high enough for the gut microorganisms to sequester sufficient amounts for growth and metabolism; thus, they may not be dependent on intracellular AA synthesis, including aromatic AA, and glyphosate would not be expected to have an antimicrobial effect, since the shikimate pathway would then be redundant (30). This argument has been supported by observations where, compared to free-living bacteria, a high proportion of host-associated gut bacteria seem to harbor an incomplete shikimate pathway (34, 48) and/or a shikimate pathway, where the genes are only transcribed to a limited degree in the gut environment (34). On the other hand, intermediates of the shikimate pathway (shikimate and 3-dehydroshikimic acid) were observed to accumulate in the ceca of rats fed diets amended with pure glyphosate or a GBH (Roundup MON 52276), indicating the inhibition of active EPSPS within the gut microbiota (44).

### Conclusion.

We observed glyphosate-associated changes in the gastrointestinal microbial ecology of piglets, which for 5 weeks from weaning were fed diets spiked with glyphosate levels equal to or 10-fold greater than the EU-defined maximum residue levels (MRLs) for common feed crops. The digesta glyphosate level increased along the gastrointestinal tract, reaching concentrations in the colon similar to those of the respective diets and comparable to the MICs reported for certain gut bacteria. However, we observed no clear glyphosate dose response of the changes and no indication of glyphosate-associated dysbiosis leading to postweaning bloom in pathogens, such as enterotoxigenic E. coli. We mainly observed changes of the commensal microbiota in the distal gut segments after 35 days of exposure to glyphosate-spiked diets. We did not analyze the digesta AA levels in the present study. However, this is a crucial factor when evaluating and discussing the potential effects of glyphosate on the gastrointestinal microbial ecology of both animals and humans.

## MATERIALS AND METHODS

### Ethics statement.

The animal experiment was conducted according to the protocol (2017-15-0201-01229), approved by the Danish Animal Experiments Inspectorate, Danish Ministry of Food, Agriculture and Fisheries, Danish Veterinary and Food Administration, and was in compliance with the Danish Ministry of Justice, law number 253 (8 March 2013), concerning experiments with animals and the care of experimental animals.

### Animals, housing, and dietary treatments.

A total of 104 crossbred [Duroc × (Danish Landrace × Yorkshire)] piglets (13 litters of 8 piglets, 53 females, and 51 males) were provided from a commercial herd. At weaning (28 ± 1 days of age), piglets were transferred litterwise to the stables at AU Viborg. The piglets were weighed and housed individually. Two piglets per litter were allocated to each of four dietary treatments: CON (control, no glyphosate added), GM_20_ (20 mg/kg glyphosate as Glyphomax), and IPA_20_ (20 mg/kg glyphosate as IPA salt) and IPA_200_ (200 mg/kg glyphosate as IPA salt). We used the glyphosate isopropylamine (IPA) salt (Monsanto, St. Louis, MO) as a pure compound and Glyphomax (Albaugh UK, Ltd., London, UK) with a glyphosate concentration of 480 g/ L as a commercial product. The adjuvants used in this formulation were kept confidential by the company, but it was not polyethoxylated tallow amine (polyoxyethyleneamine [POEA]) since glyphosate products containing this adjuvant were banned in the EU as of 2016. The glyphosate levels were chosen to investigate the influence of dietary glyphosate at a concentration (20 mg/kg) similar to the MRL set by the EU for common feed crops such as soybeans, oat, and barley, and we used a concentration 10-fold higher to investigate a level beyond this MRL. GBHs typically contain adjuvants that may influence the biological activity of glyphosate or may themselves possess biological (e.g., antimicrobial) activity; thus, a commercial GBH (Glyphomax) was included, recognizing that the nature and concentration of the adjuvants may differ from product to product (42, 49). The piglets were fed *ad libitum* with a weaning diet (Tables 1 and 2) and had free access to water through nipple drinkers. Feed intake, fecal scores, and body weight were monitored as outlined by and reported in Krogh et al. (37).

### Sampling.

Piglets were slaughtered and sampled at two time points, 9 and 35 days after weaning, referred to here as days 9 and day 35. Day 9 after weaning is typically in the period where dysbiosis and postweaning diarrhea can be encountered, whereas the gastrointestinal microbiota should have stabilized 5 weeks after weaning (50). For practical reasons, the first slaughter and sampling had to be performed over 2 days (after 9 and 10 days in treatment). We will, however, refer to this time point as day 9. On the sampling days, the barn light was turned on 3 h before slaughter to trigger the piglets to eat and thereby increase the probability for digesta to be present in the gastrointestinal tract at slaughter. The piglets were sacrificed by captive bolt stunning and bleeding. The gut was removed immediately and divided into the following eight segments: stomach, three equal-length segments of the small intestine (Si1, Si2, and Si3), cecum, and three equal-length segments of the colon (Co1, Co2, and Co3). Digesta from four of these segments, i.e., the stomach, small intestine (Si3), cecum, and colon (Co2), were selected for further analysis. The total weight and pH of the digesta were recorded. Digesta subsamples for dry matter (DM), glyphosate, AMPA, and organic acid (SCFA and lactate) analyses were immediately placed on ice and stored at −20°C until further analysis. For microbiota analysis, digesta were snap-frozen in liquid nitrogen and stored at −80°C until further analysis.

### Analytical methods (SCFA, pH, dry matter, and glyphosate).

SCFAs and lactate were analyzed by capillary gas chromatography as described by Jensen et al. (51), with modifications described by Canibe et al. (52). The pH of the digesta was measured with a combined glass/reference electrode. Dry matter content was determined by freeze-drying the digesta samples. Glyphosate and the degradation product AMPA were analyzed in feed and digesta by a micro-liquid chromatography-tandem mass spectrometry method as described by Nørskov et al. (53).

### DNA extraction and 16S rRNA gene amplicon sequencing.

DNA was extracted from 200 mg of the digesta samples using an E.Z.N.A. stool DNA kit (Omega Bio-Tek, Norcross, GA) according to the manufacturer’s instructions. The 16S rRNA amplicons were prepared for MiSeq compositional sequencing according to the Illumina 16S metagenomics sequencing library preparation protocol, with some modification as described by Tawakoli et al. (54). The universal bacterial primers Bac 341F (CCT ACG GGN GGC WGC AG) and Bac 805R (GAC TAC HVG GGT ATC TAA TCC) were used to amplify the V3-V4 regions in the first PCR amplification (20 cycles). The same primer set, with overhang adaptors, was used in the second PCR amplification (10 cycles). The third PCR amplification (eight cycles) was carried out using Nextera XT index primers (Illumina, San Diego, CA). The total PCR volume was 25 μL, using 2.5 μL of DNA sample, 0.5 μL of each primer, and 12.5 μL of 2× KAPA HiFi Hotstart ReadyMix; we finally added distilled water up to the volume (Kapa Biosystems, Boston, MA). All PCR amplifications were carried out on a Veriti 96-well thermal cycler (Applied Biosystems, Foster City, CA). AMPure XP beads (Illumina) were used after every PCR for purification of the amplified product. Quant-iT HS reagents (Molecular Probes, Eugene, OR) were used to measure the DNA concentration according to the manufacturer’s instructions. Finally, the samples were diluted to 3 ng of DNA/μL and pooled. Sequencing of the pooled samples was done on an Illumina MiSeq desktop sequencer, using 300-bp paired-end chemistry, according to the manufacturer’s instructions.

### Bioinformatic analysis of sequence data.

The Quantitative Insights Into Microbial Ecology pipeline (QIIME 1.9.1) (55) was used for processing of the MiSeq sequencing raw data. The forward and reverse *fastq* files were joined using the Python script *multiple_join_paired_ends.py* (55). After joining reads, data quality control was performed using the Python script *split_libraries_fastq.py* and the sequences were filtered with a Phred score cutoff 20 (56). OTUs were identified by the Python script *pick_open_reference_otus.py* with a subsampling percentage of 10% as proposed by Rideout et al. (57). After use of the subsampled open-reference OTU calling approach, chimeras were identified and removed with BLAST utilizing QIIME (58). Lastly, to obtain a final OTU data set, filtering out of singletons and OTUs, representing less than 0.005% of the total counts, was performed according to the method of Bokulich et al. (56). Taxonomy was assigned with QIIME, using the Greengenes 13.8 taxonomic database (59). These OTUs were agglomerated into phyla and genera using the *tax_glom* function of the PhyloSeq package in R (Fig. 1 and 2) (60).

### Statistical analyses.

**(i) Microbial diversity.** The diversity and the relative abundance of the gut microbiota were analyzed in R 3.5.1 (61). The alpha-diversity (Shannon index and richness) and beta-diversity (Whittaker and Bray-Curtis dissimilarity indices) were calculated with the Vegan R package v2.5-6 (62); both indices are quantitative and nonphylogenetic diversity metrics (63, 64). The Shannon index was estimated by using the *diversity* function of Vegan, and the differences in richness and Shannon index were examined by analysis of variance (ANOVA) using Vegan. The potential effects of the factors (treatment, sampling day, gut segment, and sex) involved in the study were examined by using the *aov* function in R (Table 5; see also Table S1 and Fig. S1 and S2 in the supplemental material). The Whitaker indices of beta-diversity were estimated using the *betadiver* function from Vegan. The homogeneity of beta-diversity among groups (given by the factor treatment, sampling day, gut segment, and sex) were then assessed by analysis of variance using the *betadisper* function from Vegan, where multivariate dispersions (variances) were calculated by an average distance of group members to the spatial median group centroid (Table 6; see also Table S1). In addition, the dissimilarities among the samples were calculated by the Bray-Curtis dissimilarity index (65), which is a quantitative (considers relative abundance), nonphylogenetic beta-diversity metric (66). Finally, these results were visualized by nonlinear multidimensional scaling (NMDS) using Vegan (see Fig. S1C and S2C). We used the *envfit* function in Vegan (999 permutations) to evaluate the significance of the association between the tested factors and the fitted NMDS ordinations.

PICRUSt software (67) was used with KEGG (68) orthologs (KOs) for the functional predictions of the 16S rRNA sequences by removing the OTUs not present in the Greengenes 13.5 database (59). NMDS plots of the generated KO abundances were produced with Vegan as described above. In addition to this, the PICRUSt Python script *categorize_by_function.py* was run in QIIME 1.9.1 to collapse the KOs at pathway levels (see Fig. S3).

**(ii) SCFA, pH, DM, glyphosate, AMPA, and microbial abundances.** The effect of treatment (glyphosate-spiked feed) was analyzed in R and conducted separately for segments for each day in treatment, fitting a linear mixed model with the *lme* function from the nlme R package v3.1-147 (69). Treatment and sex were included as fixed effects, and litter was included as a random effect in linear mixed models for each of the response variables (glyphosate, AMPA, pH, DM, organic acids, relative OTU abundances) in Tables 3, 4, and 6 (see also Tables S2 and S3 in the supplemental material). The assumption of normality of residuals was checked by qq-plots and the Shapiro-Wilks test, while Bartlett’s test was used to check for variance homogeneity. Due to the heterogeneity of glyphosate and AMPA, the variance was allowed to depend on treatment.

Fixed effects were tested with likelihood ratio test by removing the effect from the model and comparing with this reduced model using the anova function in R. When treatment was found to be significant (*P ≤ *0.05), the multcomp R package v1.4-8 (70) was utilized to perform selected *post hoc* tests with adjustment for multiple comparisons using the default single-step method. The three following treatment contrasts were tested: (i) the effect of 20 mg/kg glyphosate (CON versus IPA_20_); (ii) the effect of 200 mg/kg glyphosate (CON versus IPA_200_); and (iii) the effect of adjuvants in the commercial product (GM_20_ versus IPA_20_). Overall treatment effects with *P* values of ≤0.05 that cannot be attributed to one of the above-mentioned contrasts may occur due to the contrasts CON versus GM_20_, GM_20_ versus IPA_200_, and IPA_20_ versus IPA_200_. However, we do not consider these contrasts to add further information, and they will therefore not be considered and discussed.

Results are presented as estimated marginal means (EM-means). We use “*P* value” for the overall treatment effect and “*P*_adj_ value” to describe the effect of the selected treatment contrasts as defined above.

### Data availability.

Raw microbiome sequence reads are deposited in the NCBI short-read archive database under BioProject accession number PRJNA938476.

## Supplementary Material

Reviewer comments
