# Supplementary material for: Gastrointestinal Microbial Ecology of Weaned Piglets Fed Diets with Different Levels of Glyphosate
Source: Microbiol Spectr. 2023 Jun 15;11(4):e00615-23. doi: 10.1128/spectrum.00615-23 (PMC10433988; doi:10.1128/spectrum.00615-23)
Supplement: Supplemental file 1 — Tables S1 to S4 and Fig. S1 to S3. Download spectrum.00615-23-s0001.pdf, PDF file, 1.1 MB [file spectrum.00615-23-s0001.pdf]

Table S1. Microbial diversity in gastrointestinal digesta from piglets, sacrificed on day 9 or 35 of treatment. Data are presented as EM-means (n = 52 piglets per day).

**Diversity index**		**Segment**	**Day**		**SEM**[a]	***P*- value**
			9	35		
Alpha diversity	Richness	Stomach	251	243	9.55	0.01
		Small intestine (Si3)	132	148	5.90	0.05
		Caecum	404	444	7.90	<0.001
		Colon (Co2)	428	478	6.99	<0.001
	Shannon	Stomach	3.24	3.28	0.05	0.34
		Small intestine (Si3)	1.75	2.12	0.07	<0.01
		Caecum	4.29	4.55	0.05	<0.001
		Colon (Co2)	4.58	4.77	0.03	<0.001
Beta diversity	Whittaker	Stomach	0.44	0.45	0.01	0.01
		Small intestine (Si3)	0.50	0.50	0.01	0.81
		Caecum	0.41	0.37	0.01	<0.001
		Colon (Co2)	0.35	0.36	0.01	<0.001

[a]Average of standard errors of means

Table S2. Relative abundance of a selected set of bacterial phyla and genera in gastrointestinal digesta from piglets, sacrificed on day 9 of treatment. Data are presented as EM-means (n = 13 piglets per treatment).

Segment	Phylum	Genus	Treatment[a]				SEM[b]	*P*-value
			CON	GM_{20}	IPA_{20}	IPA_{200}		
Stomach	Firmicutes		65.70	63.90	63.30	61.20	4.58	0.92
		g__Turicibacter	0.01	0.00	0.01	0.00	0.00	0.34
		g__Streptococcus	0.58	1.18	1.20	0.96	0.30	0.46
		g__Lactobacillus	38.10	42.00	40.00	37.50	4.63	0.90
		g__Megasphaera	10.80	8.44	8.73	9.21	1.43	0.65
		g__Anaerovibrio	0.09	0.07	0.01	0.01	0.04	0.47
		g__Oscillospira	0.11	0.11	0.12	0.09	0.03	0.91
		g__Ruminococcus	0.05	0.03	0.04	0.03	0.01	0.46
		g__Coprococcus	0.06	0.02	0.01	0.03	0.01	0.43
		g__Roseburia	0.45	0.69	0.61	0.51	0.23	0.90
		g__Blautia	0.07	0.03	0.07	0.06	0.02	0.78
		f__Clostridiaceae; g__	0.02	0.25	0.04	0.11	0.05	0.03[#]
		f__Ruminococcaceae ; g__.38	0.29	0.22	0.30	0.36	0.09	0.74
		o__Clostridiales ; g__.39	1.31	1.06	0.92	0.98	0.31	0.81
		f__Lachnospiraceae ; g__.40	2.23	1.18	1.60	1.39	0.46	0.44
	Proteobacteria		11.90	15.20	13.30	18.00	3.63	0.66
		f__Enterobacteriaceae ; g__.5	0.07	0.30	0.33	1.15	0.31	0.08
		g__Actinobacillus	1.29	3.19	3.17	4.50	1.16	0.28
		g__Succinivibrio	0.21	0.44	0.07	0.26	0.24	0.75
		g__Desulfovibrio	0.21	0.35	0.29	0.22	0.12	0.80
		g__Treponema	0.06	0.18	0.08	0.22	0.08	0.46
		g__Campylobacter	5.98	5.85	4.41	4.29	1.65	0.82
	Bacteroidetes		10.19	8.38	10.00	8.63	1.52	0.78
		g__Prevotella 1	8.42	6.25	7.14	6.60	1.56	0.78
		f__ Paraprevotellaceae; g__CF231	0.00	0.00	0.01	0.01	0.00	0.43
		f__ Paraprevotellaceae; g__[Prevotella] 2	0.24	0.66	0.54	0.56	0.29	0.77
		o__Bacteroidales 2 ; g__.24	0.20	0.25	0.17	0.22	0.10	0.90
	Cyanobacteria		0.60	1.38	1.34	1.04	0.40	0.51
	Spirochaetes	g__Treponema	0.06	0.18	0.08	0.22	0.08	0.46
	Fusobacteria	g__Fusobacterium	0.20	1.22	0.65	0.66	0.42	0.43
Small intestine (Si3)	Firmicutes		75.50	77.50	80.60	74.70	4.76	0.83
		g__Turicibacter	1.02	0.45	0.84	0.19	0.60	0.75
		g__Streptococcus	0.48	0.99	1.32	1.08	0.39	0.51
		g__Lactobacillus	62.60	64.20	67.90	62.60	6.31	0.90
		g__Megasphaera	5.06	2.83	4.30	4.24	1.09	0.54
		g__Anaerovibrio	0.00	0.01	0.03	0.01	0.01	0.40
		g__Oscillospira	0.01	0.01	0.01	0.02	0.00	0.34
		g__Ruminococcus	0.38	0.05	0.04	0.20	0.13	0.28
		g__Coprococcus	0.01	0.02	0.03	0.01	0.01	0.52
		g__Roseburia	0.00	0.03	0.01	0.01	0.01	0.62
		g__Blautia	0.02	0.04	0.03	0.04	0.01	0.75
		f__Clostridiaceae; g__	1.79	2.09	1.49	2.12	1.23	0.98
		g__Dorea	0.01	0.02	0.01	0.02	0.01	0.34
		f__Ruminococcaceae ; g__.38	0.05	0.15	0.10	0.11	0.04	0.50
		o__Clostridiales ; g__.39	0.09	0.16	0.09	0.10	0.04	0.68
		f__Lachnospiraceae ; g__.40	0.18	0.25	0.45	0.22	0.11	0.35
	Proteobacteria		17.40	17.80	11.80	20.10	4.69	0.65
		f__Enterobacteriaceae ; g__.5	12.40	16.10	11.00	18.90	4.68	0.60
		g__Actinobacillus	5.34	0.43	0.84	0.60	1.62	0.11
		g__Succinivibrio	0.00	0.00	0.01	0.00	0.00	0.41
		g__Desulfovibrio	0.01	0.02	0.01	0.01	0.01	0.67
		g__Treponema	0.00	0.01	0.00	0.00	0.00	0.30
		g__Campylobacter	0.24	2.73	0.19	0.32	1.36	0.48
	Bacteroidetes		0.09	0.08	0.07	0.08	0.02	0.96
		g__Prevotella 1	0.02	0.01	0.01	0.01	0.01	0.51
		f__ Paraprevotellaceae; g__CF231	0.00	0.01	0.00	0.00	0.00	0.99
		f__ Paraprevotellaceae; g__[Prevotella] 2	0.01	0.01	0.01	0.01	0.01	0.77
		o__Bacteroidales 2 ; g__.24	0.00	0.01	0.01	0.01	0.00	0.52
	Fusobacteria	g__Fusobacterium	0.05	0.01	0.01	0.30	0.15	0.48
	Cyanobacteria		0.04	0.01	0.01	0.01	0.01	0.09
Caecum	Firmicutes		63.80	61.10	65.90	65.00	4.00	0.83
		g__Dorea	0.89	1.61	0.50	1.87	0.44	0.10
		g__Lachnospira	0.41	0.53	0.69	0.30	0.13	0.24
		g__Turicibacter	0.09	0.11	0.15	0.11	0.06	0.90

		g__Streptococcus	0.13	0.37	0.28	0.14	0.11	0.40
		g__Lactobacillus	15.80	16.40	15.60	17.40	3.17	0.97
		g__Phascolarctobacterium	1.47	1.43	1.88	1.48	0.34	0.74
		g__Megasphaera	2.31	1.98	3.68	1.31	0.90	0.27
		g__Anaerovibrio	1.18	3.03	1.94	1.78	0.63	0.20
		g__Oscillospira	1.17	1.14	1.14	1.42	0.18	0.60
		g__Ruminococcus	1.58	1.59	1.83	1.60	0.32	0.92
		g__Coprococcus	2.86	1.82	2.16	1.82	0.41	0.25
		g__Roseburia	2.32	1.39	2.03	2.12	0.56	0.67
		g__Blautia	2.02	1.96	2.33	2.50	0.42	0.77
		f__Clostridiaceae; g__	0.48	0.50	0.41	0.43	0.13	0.90
		f__Ruminococcaceae ; g__.38	15.60	11.00	13.20	14.20	1.12	0.04
		o__Clostridiales ; g__.39	4.52	4.23	4.54	4.02	0.80	0.90
		f__Lachnospiraceae ; g__.40	7.25	6.95	8.82	6.64	0.97	0.36
	Proteobacteria		12.32	14.06	9.92	12.86	2.45	0.65
		g__Campylobacter	4.56	5.77	3.25	5.54	1.34	0.50
		f__Enterobacteriaceae ; g__.5	4.16	4.65	4.52	5.62	1.87	0.94
		g__Actinobacillus	0.45	0.33	0.36	0.08	0.21	0.61
		g__Succinivibrio	0.72	0.58	0.40	0.23	0.38	0.80
		g__Desulfovibrio	0.80	0.83	0.73	0.63	0.14	0.72
	Bacteroidetes		20.40	20.30	20.00	18.20	2.48	0.89
		o__Bacteroidales 2 ; g__.24	4.69	2.90	2.51	3.39	0.86	0.30
		f__ Paraprevotellaceae; g__[Prevotella] 2	3.64	3.27	6.15	4.36	0.80	0.06[#]
		g__Prevotella 1	2.35	4.19	3.53	2.87	0.93	0.51
		f__ Paraprevotellaceae; g__CF231	2.01	1.79	1.49	1.29	0.42	0.61
		o__Bacteroidales; f__RF16 ; g__.17	1.43	1.40	0.68	0.86	0.55	0.68
		o__Bacteroidales 1 ; g__.19	0.02	0.07	0.06	0.08	0.03	0.67
	Cyanobacteria		0.03	0.19	0.01	0.03	0.04	0.03[#]
	Fusobacteria	g__Fusobacterium	0.01	0.02	0.00	0.07	0.02	0.50
	Spirochaetes	g__Treponema	1.29	2.02	1.67	1.47	0.60	0.83
Colon (Co2)	Firmicutes		62.50	61.80	61.40	62.70	3.31	0.99
		f__Ruminococcaceae ; g__.38	16.20	14.30	15.30	15.20	1.15	0.70
		g__Lactobacillus	12.10	12.80	10.80	12.00	2.43	0.94
		f__Lachnospiraceae ; g__.40	6.99	6.42	7.42	7.15	0.73	0.80
		o__Clostridiales ; g__.39	5.31	5.92	4.96	5.25	0.83	0.80
		g__Dorea	1.23	2.09	0.77	1.68	0.44	0.18
		g__Lachnospira	0.31	0.35	0.52	0.30	0.10	0.41
		g__Turicibacter	0.14	0.11	0.07	0.04	0.06	0.70
		g__Streptococcus	0.10	0.40	0.17	0.24	0.13	0.43
		g__Phascolarctobacterium	1.85	1.77	2.26	1.75	0.41	0.79
		g__Megasphaera	1.61	1.44	2.64	2.31	0.76	0.64
		g__Anaerovibrio	1.06	0.88	1.35	1.17	0.38	0.84
		g__Oscillospira	1.72	2.26	1.44	1.74	0.28	0.24
		g__Ruminococcus	1.76	1.88	1.98	1.90	0.28	0.90
		g__Coprococcus	2.99	2.16	2.46	2.13	0.51	0.61
		g__Roseburia	1.50	0.98	1.93	1.48	0.37	0.35
		g__Blautia	2.20	1.51	2.02	1.70	0.34	0.49
		f__Clostridiaceae; g__	0.70	0.87	0.44	0.55	0.22	0.57
	Proteobacteria		6.00	6.62	5.50	7.26	1.39	0.82
		f__Enterobacteriaceae; g__.5	1.91	1.85	2.16	2.87	0.95	0.86
		g__Desulfovibrio	1.51	1.67	1.07	1.17	0.19	0.10
		g__Campylobacter	1.37	1.66	1.19	1.96	0.55	0.77
		g__Actinobacillus	0.21	0.09	0.04	0.07	0.08	0.54
		g__Succinivibrio	0.05	0.14	0.26	0.07	0.06	0.13
	Bacteroidetes		25.50	25.70	27.30	24.90	2.52	0.91
		o__Bacteroidales 2 ; g__.24	5.69	4.71	4.18	4.45	0.64	0.38
		g__Prevotella 1	3.65	4.66	6.57	3.79	0.96	0.13
		f__ Paraprevotellaceae; g__[Prevotella] 2	2.87	3.46	5.50	4.19	0.77	0.10
		f__ Paraprevotellaceae; g__CF231	2.27	2.67	1.93	1.52	0.57	0.53
		o__Bacteroidales; f__RF16 ; g__.17	1.41	0.71	0.41	0.95	0.45	0.46
		o__Bacteroidales 1 ; g__.19	0.03	0.05	0.06	0.11	0.03	0.55
	Cyanobacteria		0.04	0.11	0.03	0.04	0.02	0.19
	Fusobacteria		0.01	0.08	0.00	0.08	0.05	0.50
		g__Fusobacterium	0.01	0.08	0.00	0.08	0.05	0.51
	Spirochaetes	g__Treponema	2.91	2.26	2.58	2.11	0.65	0.83

[a]Control (CON), 20 mg/kg glyphosate as Glyphomax® (GM_{20}), 20 mg/kg glyphosate as IPA salt (IPA_{20}), 200 mg/kg glyphosate as IPA salt (IPA_{200})

[b]Average of standard errors of means

$^{\#}P_{adj} < 0.05$, $^{\#\#}P_{adj} < 0.01$, $^{\#\#\#}P_{adj} < 0.001$ (effect of Glyphomax® additives, contrast GM_{20} vs IPA_{20})

Table S3. Relative abundance of a selected set of bacterial phyla and genera in gastrointestinal digesta from piglets, sacrificed on day 35 of treatment. Data are presented as EM-means (n = 13 piglets per treatment).

**Segment**	**Phylum**	**Genus**	**Treatment**[a]				**SEM**[b]	***P*-value**
			CON	GM_{20}	IPA_{20}	IPA_{200}		
Stomach	Firmicutes		60.30	63.20	55.80	63.20	5.04	0.70
		g__Turicibacter	0.07	0.03	0.09	0.02	0.03	0.41
		g__Streptococcus	0.92	0.65	1.17	0.81	0.22	0.41
		g__Lactobacillus	35.10	39.90	43.10	43.90	5.56	0.67
		g__Megasphaera	9.42	10.39	5.62	9.58	1.50	0.10
		g__Anaerovibrio	0.04	0.02	0.05	0.07	0.03	0.72
		g__Oscillospira	0.11	0.05	0.25	0.08	0.06	0.17
		g__Ruminococcus	0.12	0.06	0.13	0.10	0.04	0.71
		g__Coprococcus	0.10	0.13	0.08	0.12	0.05	0.93
		g__Roseburia	0.26	0.31	1.15	0.14	0.39	0.26
		g__Blautia	0.13	0.21	0.17	0.48	0.18	0.54
		f__Clostridiaceae; g__	0.80	0.35	0.61	0.32	0.21	0.35
		f__Ruminococcaceae ; g__.38	0.78	0.96	0.88	1.08	0.44	0.97
		o__Clostridiales ; g__.39	0.36	0.30	0.36	0.33	0.10	0.97
		f__Lachnospiraceae ; g__.40	0.97	1.07	0.86	0.70	0.28	0.79
		g__Dorea	0.13	0.17	0.17	0.23	0.10	0.93
	Proteobacteria		18.10	19.30	29.30	19.60	4.31	0.24
		f__Enterobacteriaceae ; g__.5	0.88	0.14	1.18	0.81	0.55	0.60
		g__Actinobacillus	2.83	1.89	4.15	2.89	0.70	0.17
		g__Succinivibrio	0.21	0.44	0.07	0.26	0.24	0.75
		g__Desulfovibrio	0.05	0.06	0.03	0.06	0.03	0.93
		g__Treponema	0.07	0.01	0.05	0.06	0.03	0.56
		g__Campylobacter	2.93	4.88	5.00	3.88	1.78	0.80
	Bacteroidetes		8.82	7.88	3.79	5.50	1.62	0.12
		g__Prevotella 1	8.68	8.09	3.26	5.15	1.72	0.10
		f__ Paraprevotellaceae; g__CF231	0.03	0.01	0.02	0.03	0.01	0.78
		f__ Paraprevotellaceae; g__[Prevotella] 2	0.08	0.07	0.17	0.06	0.03	0.07
		o__Bacteroidales 2 ; g__.24	0.12	0.11	0.07	0.12	0.05	0.91
	Cyanobacteria		3.98	3.13	6.32	4.95	1.53	0.50
	Fusobacteria	g__Fusobacterium	0.20	1.22	0.65	0.66	0.42	0.43
	Spirochaetes	g__Treponema	0.06	0.18	0.08	0.22	0.08	0.46
Small intestine (Si3)	Firmicutes		84.70	87.20	83.80	82.20	3.41	0.76
		g__Lactobacillus	53.30	46.20	54.40	51.60	6.48	0.82
		f__Clostridiaceae; g__	20.10	27.10	18.80	21.10	4.63	0.60
		g__Megasphaera	4.30	3.31	1.66	1.80	1.09	0.28
		g__Turicibacter	1.12	2.59	1.42	2.27	0.63	0.32
		g__Streptococcus	0.79	3.45	2.65	1.48	1.04	0.28
		g__Dorea	0.01	0.02	0.04	0.04	0.02	0.60
		g__Lachnospira	0.01	0.00	0.01	0.00	0.00	0.70
		g__Anaerovibrio	0.00	0.00	0.00	0.00	0.00	0.29
		g__Oscillospira	0.00	0.00	0.01	0.01	0.00	0.11
		g__Ruminococcus	0.01	0.10	0.07	0.02	0.01	0.02[*#]
		g__Coprococcus	0.01	0.01	0.03	0.02	0.01	0.33
		g__Roseburia	0.01	0.01	0.04	0.01	0.01	0.04[#]
		g__Blautia	0.04	0.05	0.11	0.14	0.05	0.51
		f__Ruminococcaceae ; g__.38	0.13	0.19	0.23	0.21	0.08	0.80
		o__Clostridiales ; g__.39	0.10	0.17	0.23	0.09	0.07	0.53
		f__Lachnospiraceae ; g__.40	0.12	0.11	0.17	0.12	0.05	0.80
		g__Phascolarctobacterium	0.00	0.00	0.01	0.00	0.00	0.49
	Proteobacteria		7.35	7.95	13.11	13.94	3.32	0.38
		f__Enterobacteriaceae ; g__.5	5.27	3.08	5.52	2.64	2.76	0.84
		g__Actinobacillus	2.65	4.74	7.34	11.29	2.54	0.10
		g__Campylobacter	0.27	0.13	0.26	0.29	0.16	0.88
		g__Succinivibrio	0.00	0.00	0.00	0.00	0.00	0.90
		g__Desulfovibrio	0.00	0.00	0.01	0.01	0.00	0.59
	Bacteroidetes		0.12	0.07	0.11	0.17	0.04	0.54
		g__Prevotella 1	0.04	0.02	0.05	0.11	0.04	0.57
		f__ Paraprevotellaceae; g__CF231	0.01	0.00	0.00	0.01	0.00	0.31
		f__ Paraprevotellaceae; g__[Prevotella] 2	0.01	0.01	0.01	0.01	0.00	0.80
		o__Bacteroidales; f__RF16 ; g__.17	0.00	0.01	0.00	0.00	0.00	0.24
		o__Bacteroidales 1 ; g__.19	0.00	0.00	0.00	0.00	0.00	0.06
		o__Bacteroidales 2 ; g__.24	0.01	0.00	0.01	0.01	0.00	0.20
	Cyanobacteria		0.02	0.05	0.01	0.03	0.01	0.40
	Fusobacteria	g__Fusobacterium	0.03	0.02	0.02	0.01	0.01	0.80
	Spirochaetes	g__Treponema	0.00	0.00	0.00	0.01	0.00	0.82
Caecum	Firmicutes		66.00	71.30	75.00	74.00	2.84	0.13
		f__Ruminococcaceae ; g__.38	13.00	15.30	15.00	16.40	1.01	0.10
		f__Lachnospiraceae ; g__.40	11.40	10.40	10.00	9.59	1.18	0.72

		g__Lactobacillus	10.70	12.50	13.30	14.50	2.14	0.64
		o__Clostridiales ; g__.39	6.04	6.30	7.77	6.61	0.63	0.25
		g__Phascolarctobacterium	1.48	1.45	1.73	1.21	0.26	0.60
		g__Megasphaera	1.62	2.34	3.44	4.28	0.83	0.13
		g__Anaerovibrio	3.10	3.79	2.68	2.88	0.57	0.55
		g__Oscillospira	1.65	1.28	1.37	1.17	0.13	0.09
		g__Ruminococcus	2.20	2.20	2.29	1.83	0.27	0.65
		g__Coprococcus	2.07	1.81	1.58	1.50	0.22	0.28
		g__Roseburia	2.39	1.62	3.12	1.84	0.53	0.21
		g__Blautia	1.29	1.52	1.67	1.64	0.20	0.56
		f__Clostridiaceae;g__	1.70	2.42	2.16	2.16	0.74	0.92
		g__Dorea	0.85	0.84	0.86	1.09	0.14	0.26
		g__Lachnospira	1.22	1.88	1.27	1.59	0.21	0.34
		g__Turicibacter	0.12	0.22	0.32	0.23	0.07	0.37
		g__Streptococcus	0.37	0.33	0.82	0.77	0.22	0.26
	Proteobacteria		4.20	5.98	2.91	3.19	1.16	0.25
		g__Campylobacter	1.11	2.35	0.84	0.13	0.65	0.12
		g__Actinobacillus	0.09	0.40	0.32	0.77	0.23	0.24
		g__Succinivibrio	0.90	0.66	0.46	0.32	0.34	0.65
		g__Desulfovibrio	0.54	0.50	0.36	0.42	0.12	0.71
		f__Enterobacteriaceae ; g__.5	0.35	1.21	0.64	0.47	0.55	0.70
	Bacteroidetes		26.10	20.10	19.20	20.90	2.15	0.11
		g__Prevotella 1	6.78	5.66	5.66	5.55	1.07	0.80
		f__ Paraprevotellaceae; g__[Prevotella] 2	5.44	5.22	4.55	5.73	0.78	0.75
		f__ Paraprevotellaceae; g__CF231	3.71	2.02	2.33	2.07	0.40	0.01*¤
		o__Bacteroidales 2 ; g__.24	3.69	1.89	2.07	1.75	0.42	0.01*¤
		f__RF16 ; g__.17	1.16	0.48	0.43	0.34	0.42	0.50
		o__Bacteroidales 1 ; g__.19	0.05	0.05	0.08	0.08	0.02	0.70
	Cyanobacteria		0.21	0.39	0.14	0.34	0.13	0.57
	Fusobacteria	g__Fusobacterium	0.00	0.01	0.00	0.01	0.00	0.68
	Spirochaetes	g__Treponema	2.05	0.91	1.27	0.29	0.48	0.08¤
Colon (Co2)	Firmicutes		57.70	66.50	69.40	66.10	2.53	0.01*¤
		f__Ruminococcaceae ; g__.38	12.70	14.30	14.60	14.40	0.77	0.30
		f__Lachnospiraceae ; g__.40	8.11	8.88	8.00	7.77	0.84	0.80
		g__Lactobacillus	8.07	10.96	10.52	11.38	1.25	0.25
		o__Clostridiales ; g__.39	6.37	6.11	7.66	6.20	0.70	0.30
		g__Ruminococcus	2.20	2.04	1.88	1.66	0.21	0.34
		g__Phascolarctobacterium	1.37	1.71	1.36	1.42	0.25	0.73
		g__Megasphaera	1.37	3.30	3.16	4.39	0.78	0.06
		g__Anaerovibrio	1.91	2.12	2.03	2.09	0.35	0.90
		g__Oscillospira	1.78	1.79	2.03	1.62	0.14	0.20
		g__Coprococcus	1.59	1.88	1.47	1.44	0.31	0.75
		g__Roseburia	1.62	1.42	2.78	1.79	0.50	0.27
		g__Blautia	1.19	1.45	1.78	1.36	0.22	0.32
		f__Clostridiaceae; g__	1.44	1.68	1.55	1.45	0.40	0.97
		g__Dorea	0.91	1.06	1.12	0.86	0.17	0.67
		g__Lachnospira	0.94	1.03	1.04	1.15	0.18	0.88
		g__Turicibacter	0.08	0.10	0.12	0.07	0.03	0.72
		g__Streptococcus	0.76	0.57	2.32	1.79	0.51	0.05#
	Proteobacteria		3.75	4.23	2.70	3.18	0.54	0.22
		f__Enterobacteriaceae ; g__.5	0.19	0.53	0.26	0.11	0.18	0.41
		g__Actinobacillus	0.01	0.01	0.02	0.04	0.01	0.13
		g__Succinivibrio	0.89	0.47	0.37	0.43	0.27	0.54
		g__Desulfovibrio	0.92	1.49	0.94	1.29	0.22	0.23
		g__Campylobacter	0.50	0.56	0.34	0.18	0.17	0.41
	Bacteroidetes		32.60	25.10	23.50	27.40	2.03	0.01*
		g__Prevotella 1	8.02	6.94	5.39	7.74	1.45	0.58
		o__Bacteroidales 2 ; g__.24	7.12	4.56	4.59	4.00	0.66	0.01*¤
		f__ Paraprevotellaceae; g__CF231	4.07	2.89	2.79	2.76	0.43	0.11
		f__ Paraprevotellaceae; g__[Prevotella] 2	3.57	3.03	3.12	3.18	0.47	0.85
		o__Bacteroidales; f__RF16 ; g__.17	0.97	0.28	0.42	0.22	0.20	0.05¤
		o__Bacteroidales 1 ; g__.19	0.07	0.11	0.06	0.11	0.03	0.63
	Cyanobacteria		0.33	0.26	0.13	0.35	0.17	0.81
	Fusobacteria	g__Fusobacterium	0.01	0.00	0.00	0.01	0.01	0.27
	Spirochaetes	g__Treponema	2.88	1.22	1.88	0.76	0.71	0.18

[a]Control (CON), 20 mg/kg glyphosate as Glyphomax® (GM_{20}), 20 mg/kg glyphosate as IPA (IPA_{20}), 200 mg/kg glyphosate as IPA (IPA_{200})

[b]Standard errors of means averages

$^{*}P_{adj} < 0.05$, $^{**}P_{adj} < 0.01$, $^{***}P_{adj} < 0.001$ (effect of 20 mg/kg of glyphosate, contrast CON vs IPA_{20})

$^{¤}P_{adj} < 0.05$, $^{¤¤}P_{adj} < 0.01$, $^{¤¤¤}P_{adj} < 0.001$ (effect of 200 mg/kg of glyphosate, contrast CON vs IPA_{200})

$^{\#}P_{adj} < 0.05$, $^{\#\#}P_{adj} < 0.01$, $^{\#\#\#}P_{adj} < 0.001$ (effect of Glyphomax® additives, contrast GM20 vs IPA_{20})

Table S4. Glyphosate concentration in gastrointestinal digesta from piglets, sacrificed day 9 or 35 of treatment. Data are presented as EM-means (n = 13 piglets per treatment).

Day	Segment	Glyphosate[a]	Treatment[b]			
			CON	GM_{20}	IPA_{20}	IPA_{200}
Day 9	Stomach	Wet	0.01	1.18	2.40	15.3
		Dry	0.05	5.13	9.72	69.4
	Small intestine	Wet	0.01	1.63	2.42	13.7
		Dry	0.15	21.2	33.7	184
	Caecum	Wet	0.03	2.84	4.57	35.0
		Dry	0.35	33.7	41.4	391
	Colon	Wet	0.05	12.6	12.3	166
		Dry	0.33	71.8	74.4	981
Day 35	Stomach	Wet	0.02	2.12	1.63	21.0
		Dry	0.09	8.80	7.12	90.1
	Small intestine	Wet	0.04	6.91	6.04	81.4
		Dry	0.35	54.8	49.5	622
	Caecum	Wet	0.03	4.97	5.24	64.9
		Dry	0.25	41.1	43.7	541
	Colon	Wet	0.17	16.2	20.5	208
		Dry	0.86	83.2	105	1059

[a]Glyphosate concentration (mg/kg) calculated from digesta wet (Wet) or dry (Dry) weight.

[b]Control (CON), 20 mg/kg glyphosate as Glyphomax® (GM_{20}), 20 mg/kg glyphosate as IPA (IPA_{20}), 200 mg/kg glyphosate as IPA (IPA_{200}).

FIGURE LEGENDS (SUPPLEMENTARY)

Figure S1. Microbiota diversity based on observed OTUs in digesta samples (n = 104 per segment) from stomach (Sto), distal small intestine (Si3), caecum (Cae) and mid colon (Co2). Alpha diversity: Richness (A), Shannon index (B). Beta diversity: Non-metric multidimensional scaling (NMDS) plot based on Bray-Curtis dissimilarities (C). Significant effect of segment ($P < 0.001$) was observed for all three diversity parameters.

Figure S2. Microbiota diversity based on observed OTUs in digesta samples (n = 104 from each of the four segments, stomach, distal small intestine, caecum, and mid colon) from female (F, n = 53) and male (M, n = 51) piglets. Alpha diversity: Richness (A), Shannon index (B). Beta diversity: Non-metric multidimensional scaling (NMDS) plot based on Bray-Curtis dissimilarities (C). No significant effect of sex ($P > 0.05$) was observed.

Figure S3. Metagenome function predictions, using PICRUSt, and illustrated by NMDS ordination of the samples obtained through Bray-Curtis dissimilarity index for segment (A), including both day 9 and day 35 ($P = 0.001$), sampling day (B) for caecum samples only ($P < 0.001$), caecum samples on day 9 (C) and on day 35 (D) ($P > 0.05$ for both days), sex (E) ($P < 0.05$).

Fig. S1

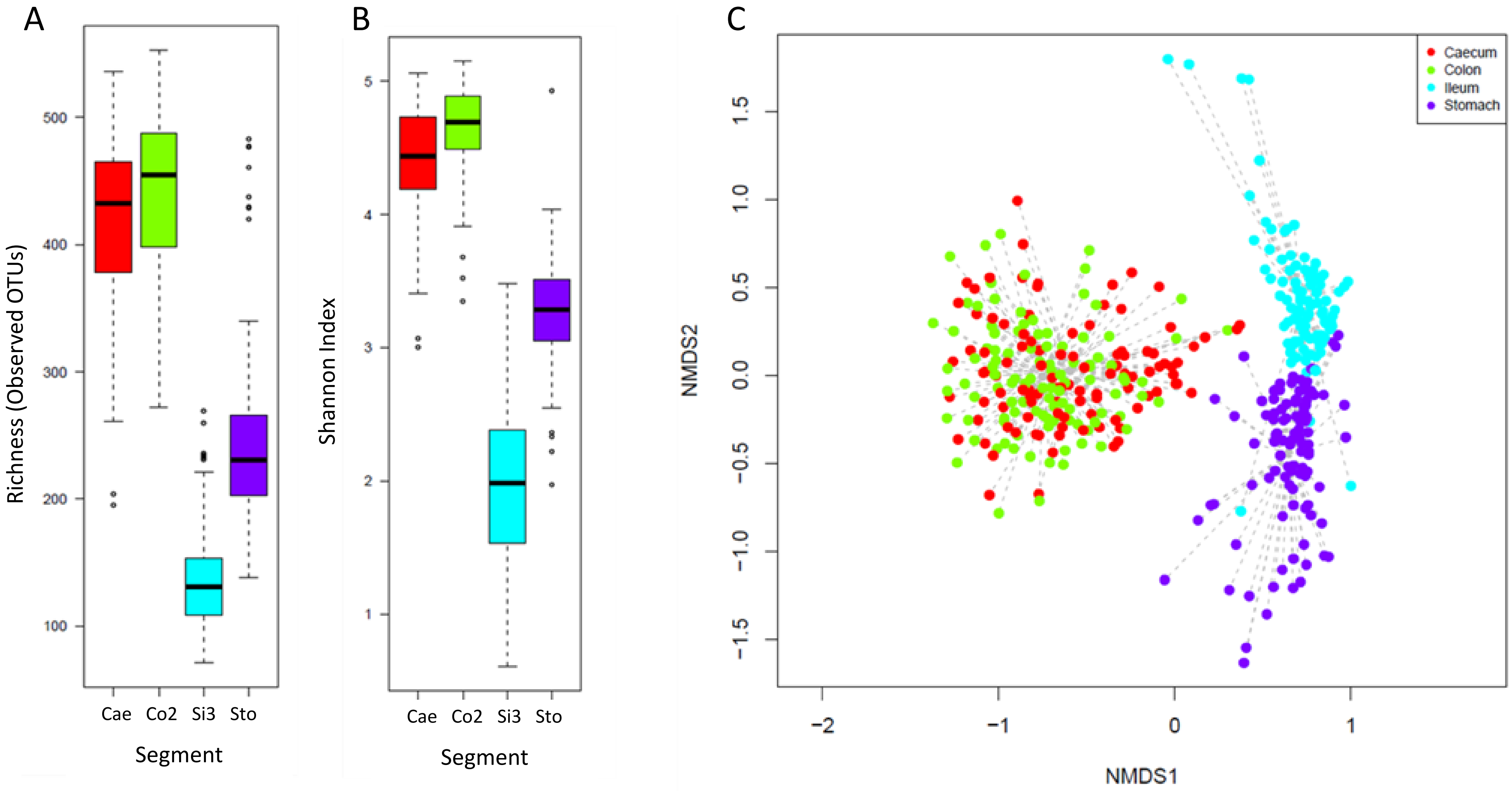
A
B
C
Richness (Observed OTUs)
100
200
300
400
500
Cae
Co2
Si3
Sto
Segment
Shannon Index
1
2
3
4
5
Cae
Co2
Si3
Sto
Segment
Caecum
Colon
Ileum
Stomach
NMDS2
−1.5
−1.0
−0.5
0.0
0.5
1.0
1.5
−2
−1
0
1
NMDS1

Fig. S2

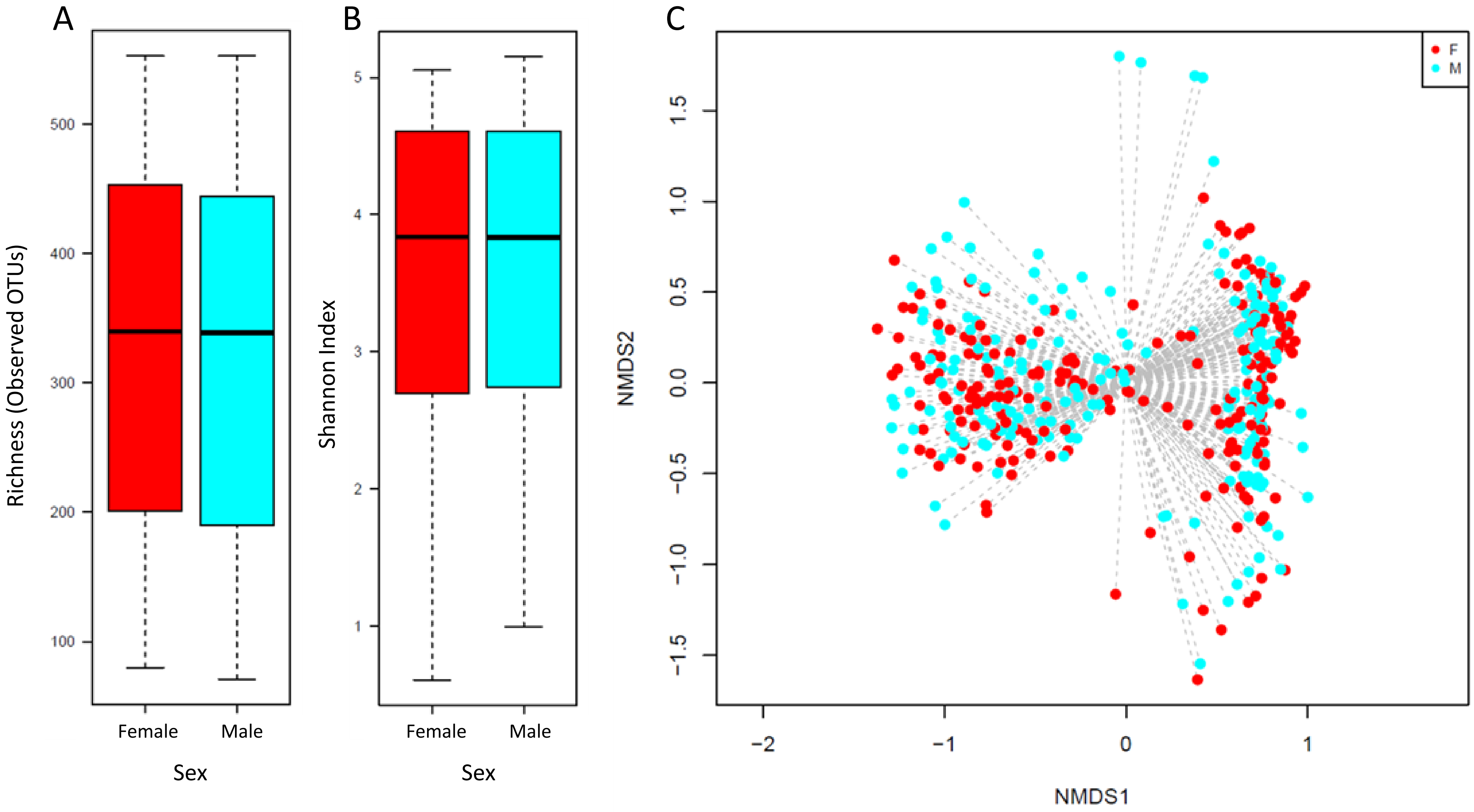
A
B
C
Richness (Observed OTUs)
100
200
300
400
500
Female
Male
Sex
Shannon Index
1
2
3
4
5
Female
Male
Sex
F
M
NMDS2
−1.5
−1.0
−0.5
0.0
0.5
1.0
1.5
−2
−1
0
1
NMDS1

Fig. S3

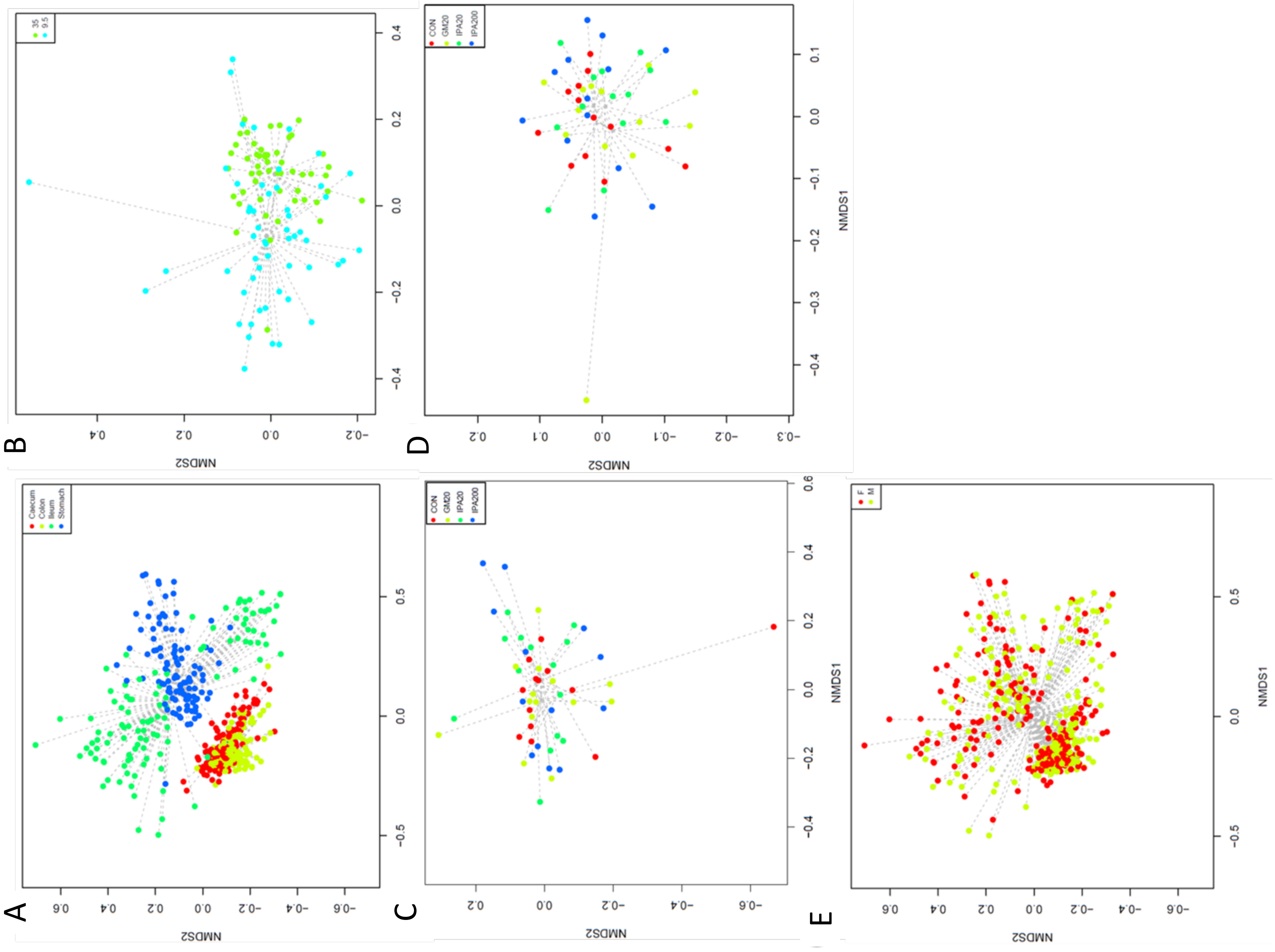

A
B
C
D
E
NMDS1
NMDS2
Caecum
Colon
Ileum
Stomach
35
9.5
CON
GM20
IPA20
IPA200
F
M
