# Supplementary material for: Gastrointestinal Microbial Ecology of Weaned Piglets Fed Diets with Different Levels of Glyphosate
Source: Microbiol Spectr. 2023 Jun 15;11(4):e00615-23. doi: 10.1128/spectrum.00615-23 (PMC10433988; doi:10.1128/spectrum.00615-23)
Supplement: Reviewer comments [file reviewer-comments.pdf]

AMERICAN SOCIETY FOR MICROBIOLOGY | Microbiology Spectrum

Gastrointestinal Microbial Ecology of Weaned Piglets Fed Diets with Different Levels of Glyphosate

Sundas Rani, Martin Sørensen, Jordi Estellé, Samantha Noel, Natalja Nørskov, Uffe Krogh, Leslie Foldager, and Ole Højberg

Corresponding Author(s): Ole Højberg, Aarhus Universitet

Review Timeline:

	Submission Date:	March 2, 2023
	Accepted:	May 26, 2023

Editor: Allison Veach

Reviewer(s): The reviewers have opted to remain anonymous.

Transaction Report:

(Note: With the exception of the correction of typographical or spelling errors that could be a source of ambiguity, letters and reports are not edited. The original formatting of letters and referee reports may not be reflected in this compilation.)

DOI: https://doi.org/10.1128/spectrum.00615-23

May 26, 2023

Dr. Ole Højberg
Aarhus Universitet
Animal and Veterinary Sciences
Blichers Alle 20
Tjele DK-8830
Denmark

Re: Spectrum00615-23 (Gastrointestinal Microbial Ecology of Weaned Piglets Fed Diets with Different Levels of Glyphosate)

Dear Dr. Ole Højberg:

Your manuscript has been accepted, and I am forwarding it to the ASM Journals Department for publication. You will be notified when your proofs are ready to be viewed.

The ASM Journals program strives for constant improvement in our submission and publication process. Please tell us how we can improve your experience by taking this quick Author Survey.

Publication Fees: We have partnered with Copyright Clearance Center to collect author charges. You will soon receive a message from no-reply@copyright.com with further instructions. For questions related to paying charges through RightsLink, please contact Copyright Clearance Center by email at ASM_Support@copyright.com or toll free at +1.877.622.5543. Hours of operation: 24 hours per day, 7 days per week. Copyright Clearance Center makes every attempt to respond to all emails within 24 hours. For a complete list of **Publication Fees**, including supplemental material costs, please visit ourwebsite.

ASM policy requires that data be available to the public upon online posting of the article, so please verify all links to sequence records, if present, and make sure that each number retrieves the full record of the data. If a new accession number is not linked or a link is broken, provide production staff with the correct URL for the record. If the accession numbers for new data are not publicly accessible before the expected online posting of the article, publication of your article may be delayed; please contact the ASM production staff immediately with the expected release date.

Corresponding authors may join or renew ASM membership to obtain discounts on publication fees. Need to upgrade your membership level? Please contact Customer Service at Service@asmusa.org.

Thank you for submitting your paper to Spectrum.

Sincerely,

Allison Veach
Editor, Microbiology Spectrum

Journals Department
American Society for Microbiology
1752 N St., NW
Washington, DC 20036
E-mail: spectrum@asmusa.org
